# IL-21 shapes germinal center polarization via light zone B cell selection and cyclin D3 upregulation

**DOI:** 10.1084/jem.20221653

**Published:** 2023-07-19

**Authors:** Lina Petersone, Chun Jing Wang, Natalie M. Edner, Astrid Fabri, Spyridoula-Angeliki Nikou, Claudia Hinze, Ellen M. Ross, Elisavet Ntavli, Yassin Elfaki, Frank Heuts, Vitalijs Ovcinnikovs, Andrea Rueda Gonzalez, Luke P. Houghton, Hannah M. Li, Yang Zhang, Kai-Michael Toellner, Lucy S.K. Walker

**Affiliations:** 1 Division of Infection and Immunity, Institute of Immunity and Transplantation, University College London, London, UK; 2 Institute of Immunology and Immunotherapy, University of Birmingham, Birmingham, UK

## Abstract

Germinal center (GC) dysregulation has been widely reported in the context of autoimmunity. Here, we show that interleukin 21 (IL-21), the archetypal follicular helper T cell (Tfh) cytokine, shapes the scale and polarization of spontaneous chronic autoimmune as well as transient immunization-induced GC. We find that IL-21 receptor deficiency results in smaller GC that are profoundly skewed toward a light zone GC B cell phenotype and that IL-21 plays a key role in selection of light zone GC B cells for entry to the dark zone. Light zone skewing has been previously reported in mice lacking the cell cycle regulator cyclin D3. We demonstrate that IL-21 triggers cyclin D3 upregulation in GC B cells, thereby tuning dark zone inertial cell cycling. Lastly, we identify Foxo1 regulation as a link between IL-21 signaling and GC dark zone formation. These findings reveal new biological roles for IL-21 within GC and have implications for autoimmune settings where IL-21 is overproduced.

## Teaser

Petersone et al. investigate IL-21–dependent germinal center (GC) regulation and reveal key roles for IL-21 in promoting light zone GC B cell selection and controlling dark zone GC B cell proliferation by upregulation of cyclin D3.

## Graphical Abstract

**Figure:**
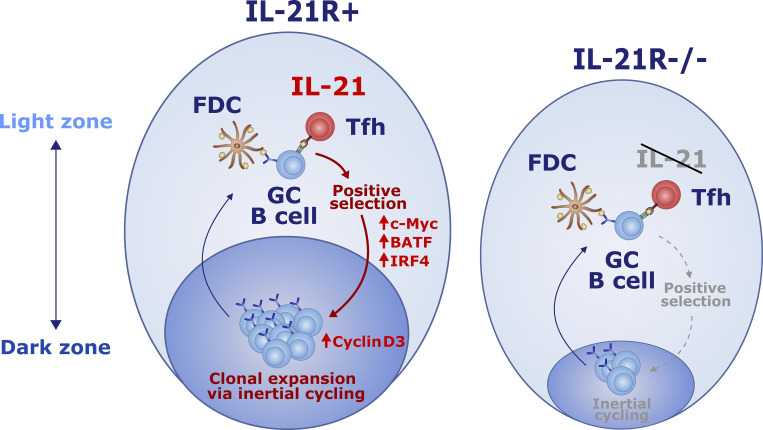

## Introduction

Germinal centers (GC) are highly dynamic tissue microenvironments that facilitate the differentiation of long-lived memory B cells and high-affinity antibody-producing plasma cells (Victora and Nussenzweig, 2022). While efficient GC responses represent a cornerstone of lasting humoral immunity against pathogens, dysregulated GC processes have been implicated in autoimmunity (Vinuesa et al., 2009).

GC formation is typically orchestrated by a specialized subset of CD4 T cells termed follicular helper T cells (Tfh) in a manner that is subject to tight regulation by the CTLA-4/CD28 axis. In the absence of CD28 signaling, CD4 T cells fail to migrate to B cell follicles and GC do not develop (Walker et al., 1999; Ferguson et al., 1996), while T cells with reduced CD28 expression as a consequence of gene heterozygosity exhibit reduced Tfh differentiation in vivo despite overtly normal proliferation (Wang et al., 2015). Conversely, mice deficient in CTLA-4 are characterized by spontaneous Tfh differentiation and the formation of large chronic GC (Wang et al., 2015), presumably due to dysregulation of CD80 and CD86 that are involved in the GC response (Salek-Ardakani et al., 2011; Good-Jacobson et al., 2012). Although the CTLA-4−/− mice exhibited global T cell activation, we found that interleukin 21 (IL-21) was the CD4 T cell–derived cytokine most overproduced in this setting of systemic autoimmunity (Wang et al., 2015). Whether elevated IL-21 is a product or driver of the humoral dysregulation in CTLA-4 deficient mice remains unclear.

IL-21 is a highly pleiotropic immune modulator with diverse functionality across a broad range of target cells (Long et al., 2019; Spolski and Leonard, 2014). Binding of IL-21 to its receptor activates Janus kinase (JAK) 1 and JAK3 signaling pathways, which facilitate downstream phosphorylation of signal transducer and activator of transcription (STAT) 1 and STAT3, and to a lesser extent STAT4 and STAT5 proteins (Asao et al., 2001; Habib et al., 2002; Strengell et al., 2003). In addition to the JAK-STAT pathways, IL-21 signaling can also activate the mitogen-activated protein kinase and phosphoinositide 3-kinase (PI3K) pathways (Zeng et al., 2007; Attridge et al., 2014). The biological impact of IL-21 signaling has been shown to be highly context-dependent with both stimulatory and proapoptotic effects (Mehta et al., 2004), making this cytokine a potent yet complex immunomodulator.

In the context of GC responses, IL-21 is the archetypal Tfh cell cytokine (Chtanova et al., 2004; Vinuesa et al., 2005) that is produced from the earliest stages of T cell dependent B cell activation (Gonzalez et al., 2018; Weinstein et al., 2016; Zhang et al., 2018). IL-21 production in GC Tfh undergoing cognate interaction with B cells is associated with calcium signaling (Shulman et al., 2014), consistent with the NFAT dependence of IL-21 expression (Kim et al., 2005), and 30–40% of Tfh are thought to be synthesizing IL-21 at any one time (Lüthje et al., 2012). Since IL-21 receptor (IL-21R) is highly expressed in Tfh cells, GC B cells, plasma cells, and at least some subsets of GC-derived memory B cells, IL-21 has been recognized as an important regulator of humoral immunity and the GC reaction (Zhang et al., 2018; Laidlaw et al., 2020; Tangye and Ma, 2020).

While IL-21 is not required for Tfh formation (Linterman et al., 2010; Zotos et al., 2010; Rasheed et al., 2013; Bessa et al., 2010), it can synergize with other signaling pathways, most notably IL-6, to enhance Tfh cell differentiation and expansion (Quast et al., 2022; Karnowski et al., 2012; Eto et al., 2011). On the other hand, B cells are known to be an important target of IL-21 signaling (Linterman et al., 2010; Zotos et al., 2010; Zhang et al., 2018; Gonzalez et al., 2018; Ozaki et al., 2002). Recent work has shown that IL-21 can promote B cell activation and expansion during the early stages of T cell–dependent immune responses (Dvorscek et al., 2022), adding to long-standing observations that mice with defective IL-21 signaling form smaller GC that dissolve more quickly (Linterman et al., 2010; Zotos et al., 2010; Zhang et al., 2018; Zotos et al., 2021). In addition to its effects on GC formation, a growing body of evidence now suggests that IL-21 can also shape B cell identity and modulate cell cycle progression within established GC (Zotos et al., 2021; Dvorscek et al., 2022; Gonzalez et al., 2018; Collins and Speck, 2015). Unsurprisingly, IL-21 has also been reported to shape GC output by promoting affinity maturation of B cell immunoglobulin genes and plasma cell differentiation (Zhang et al., 2018; Zotos et al., 2010; Linterman et al., 2010; Ozaki et al., 2004).

Consistent with data from murine models, reports describing patients with loss-of-function mutations in IL-21R–encoding genes (Erman et al., 2015; Kotlarz et al., 2013; Stepensky et al., 2015), as well as the only currently known IL-21–deficient patient (Salzer et al., 2014), have noted impaired humoral responses characterized by reduced serum IgG and elevated serum IgE levels and markedly reduced frequencies of class-switched memory B cells. Furthermore, elevated IL-21 production has been reported in patients across a wide range of autoimmune conditions with dysregulated humoral immunity, including type 1 diabetes, rheumatoid arthritis, and systemic lupus erythematosus (Kenefeck et al., 2015; Ferreira et al., 2015; Dolff et al., 2011; Terrier et al., 2012; Niu et al., 2010; Liu et al., 2012), and IL-21 has been shown to influence the course of adaptive immune responses to viral infections (Elsaesser et al., 2009; Collins and Speck, 2015). Thus, data from both murine and human studies pinpoint IL-21 as an essential regulator of humoral immunity; however, much remains to be elucidated about the exact mechanisms of IL-21–dependent GC regulation.

Given the overproduction of IL-21 in CTLA-4–deficient mice, we set out to investigate the extent to which this contributed to dysregulated GC formation by rendering these animals deficient for IL-21R. By analyzing how T cell/B cell collaboration is impacted by IL-21R deficiency in autoimmune and subsequently also immunization-induced GC, we reveal key roles for IL-21 signaling in light zone GC B cell positive selection as well as dark zone inertial cell cycling via IL-21–dependent control of cyclin D3 expression. We further identify Foxo1 as one of the pathways linking IL-21 signaling to GC dark zone formation. These findings shed light on the normal control of the GC and have implications for the dysregulation of B cell responses in autoimmune settings where IL-21 is overproduced.

## Results

### IL-21 promotes Tfh and GC B cell differentiation in CTLA-4−/− mice

**Figure 1.**
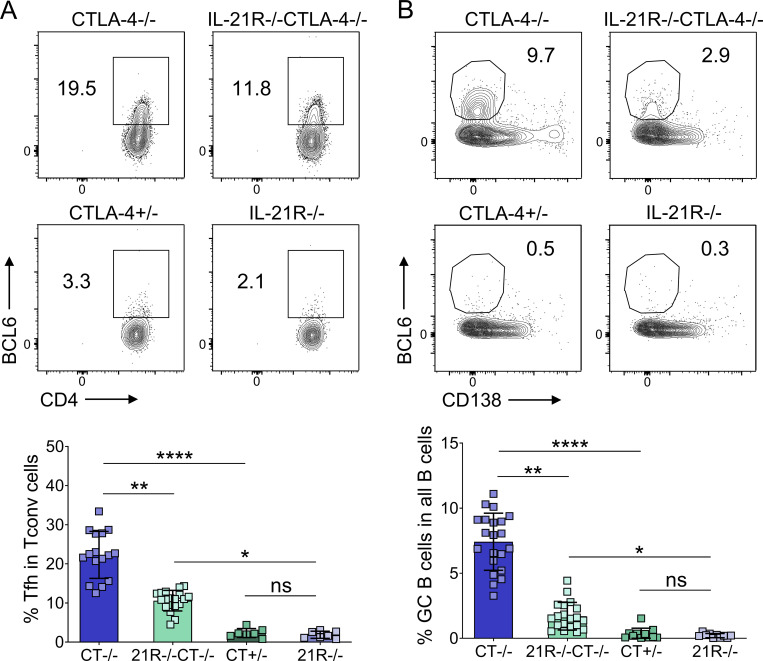
**IL-21 promotes chronic GC responses in CTLA-4−/− mice.** Splenic Tfh and GC B cells were analyzed in 17–21-d-old CTLA-4−/− and IL-21R−/−CTLA-4−/− mice and their CTLA-4+/− and IL-21R−/− littermates. **(A)** Representative flow cytometry plots (top) and collated data (bottom) showing frequency of Tfh cells (CD3+CD4+FoxP3−BCL6+) in conventional T cells (Tconv; CD3+CD4+FoxP3−). **(B)** Representative flow cytometry plots (top) and collated data (bottom) showing frequency of GC B cells (CD19+CD138−BCL-6+) in all B cells (CD19+). Data are collated from 10 independent experiments; *n* = 7–21; Kruskal–Wallis test. Mean ± SD are shown; ****, P < 0.0001; **, P < 0.01; *, P < 0.05; ns, not significant.

**Figure S1.**
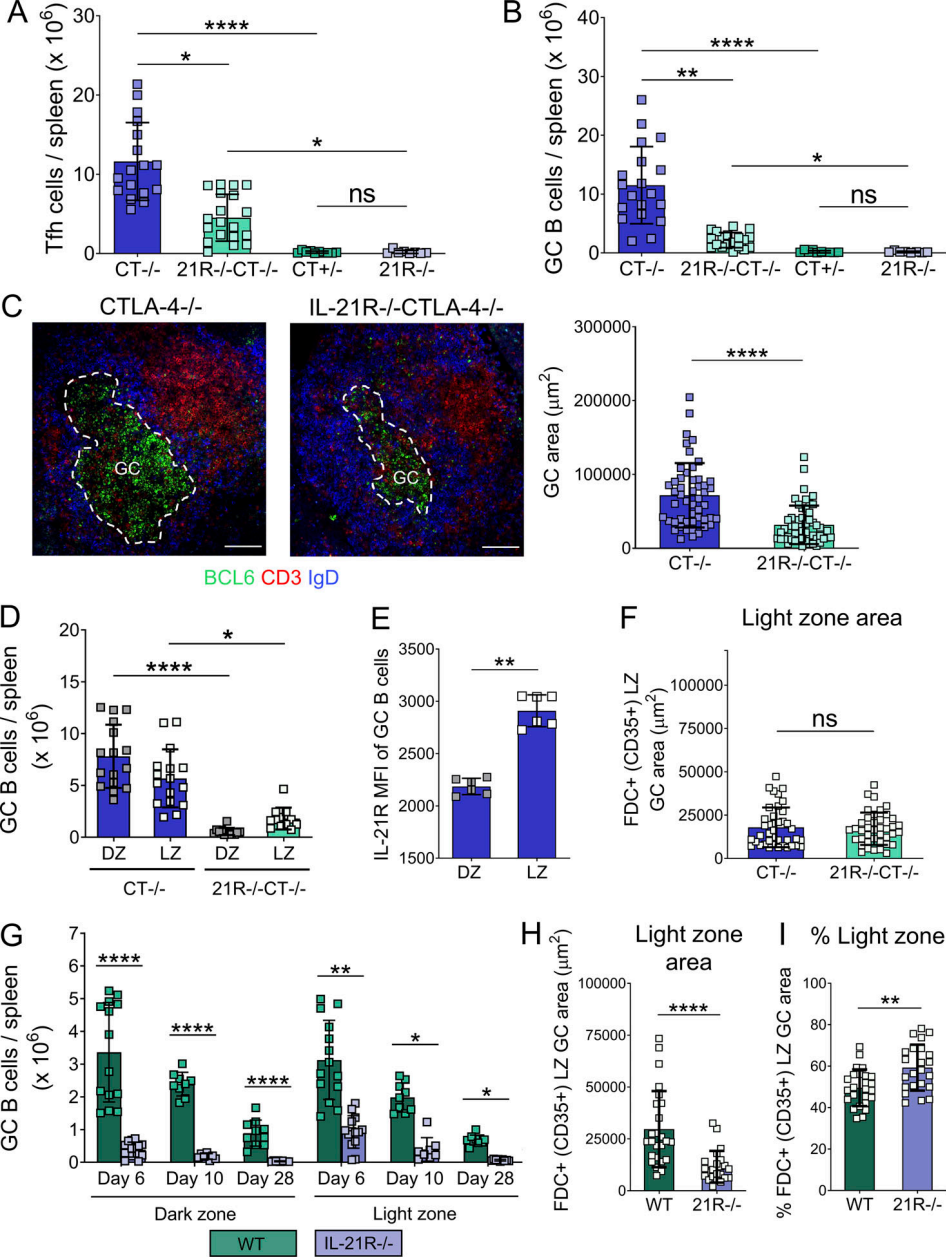
**IL-21 promotes GC expansion and polarization in CTLA-4−/− and wild-type mice.** Splenic GC were analyzed in 16–21-d-old CTLA-4−/−, IL-21R−/−CTLA-4−/− mice and CTLA-4+/−, IL-21R−/− littermates. **(A)** Collated flow cytometry data showing absolute number of Tfh cells (CD3+CD4+FoxP3−BCL6+) per spleen. **(B)** Collated flow cytometry data showing absolute number of GC B cells (CD19+CD138−BCL-6+) per spleen. Data are collated from nine independent experiments; *n* = 8–19; Kruskal–Wallis test. **(C)** Spleen sections were stained for BCL6 (green), CD3 (red), and IgD (blue). Representative confocal images (left) and collated data (right) for GC area (scale bar 100 µm; 10–11 GC/mouse, each point represents a GC). Data are collated from four independent experiments; *n* = 5; Mann–Whitney U test. **(D)** GC B cell distribution across dark zone (DZ; CXCR4^high^CD86^low^) and light zone (LZ; CXCR4^low^CD86^high^) compartments was analyzed in spleens from CTLA-4−/− and IL-21R−/−CTLA-4−/− mice. Collated data for absolute numbers of dark zone and light zone cells in the GC B cell compartment (CD19+BCL6+). Data are collated from seven independent experiments; *n* = 12–16; Kruskal–Wallis test. **(E)** Collated data for IL-21R median fluorescence intensity (MFI) in dark zone and light zone CTLA-4−/− GC B cells. Data are collated from two independent experiments; *n* = 6; Mann–Whitney U test. **(F)** GC stromal compartments were analyzed in spleen sections from CTLA-4−/− and IL-21R−/−CTLA-4−/− mice using confocal microscopy; FDC in GC light zone were identified by their CD35 expression. Collated data for light zone absolute area (CD35+) in splenic GC (10–11 GC/mouse; each dot represents a GC). Data are collated from four independent experiments; *n* = 5; Mann–Whitney U test. **(G)** GC B cell distribution across dark zone (CXCR4^high^CD86^low^) and light zone (CXCR4^low^CD86^high^) compartments was analyzed in spleens from SRBC-immunized wild-type (WT) and IL-21R−/− mice 6, 10 or 28 d after immunization. Collated data for absolute numbers of dark zone and light zone cells in the GC B cell compartment (CD19+BCL6+). Data are collated from seven independent experiments; *n* = 8–14; Kruskal–Wallis test. **(H and I)** GC compartments were analyzed in spleen sections from SRBC-immunized WT and IL-21R−/− mice 6 d after immunization using confocal microscopy. FDC in GC light zone were identified by their CD35 expression. (H) Collated data for light zone absolute area (CD35+) and (I) the percentage of GC area occupied by light zone stroma (CD35+) in splenic GC (7–9 GC/mouse; each dot represents a GC). Data are collated from three independent experiments; *n* = 3; Mann–Whitney U test. Mean ± SD are shown; ****, P < 0.0001; **, P < 0.01; *, P < 0.05; ns, not significant.

To pinpoint which aspects of the chronic GC responses in CTLA-4−/− mice were regulated by IL-21, these mice were crossed to render them deficient in IL-21R. We first compared total splenic Tfh and GC B cell populations in CTLA-4−/− and IL-21R−/−CTLA-4−/− mice and their littermate controls using flow cytometry. Tfh cells still formed in the absence of IL-21R, but their frequencies (Fig. 1 A), as well as absolute numbers (Fig. S1 A), were reduced compared with age-matched mice lacking CTLA-4 alone. The GC B cell population also showed a marked impairment in the absence of IL-21R expression, as evidenced by the significantly reduced GC B cell frequencies (Fig. 1 B) and absolute numbers (Fig. S1 B) in IL-21R−/−CTLA-4−/− mice compared with CTLA-4−/− animals. In the two sets of littermate controls, CTLA-4+/− and IL-21R−/−, Tfh, and GC B cells were present in very low numbers with no differences between the two groups. The reduced GC response in CTLA-4−/− animals lacking IL-21R expression was confirmed by confocal microscopy analysis of tissue sections (Fig. S1 C). Collectively, these data demonstrate that IL-21 signaling regulates the scale of chronic GC responses in CTLA-4−/− mice.

### CTLA-4−/− GC show impaired dark zone formation in the absence of IL-21 signaling

**Figure 2.**
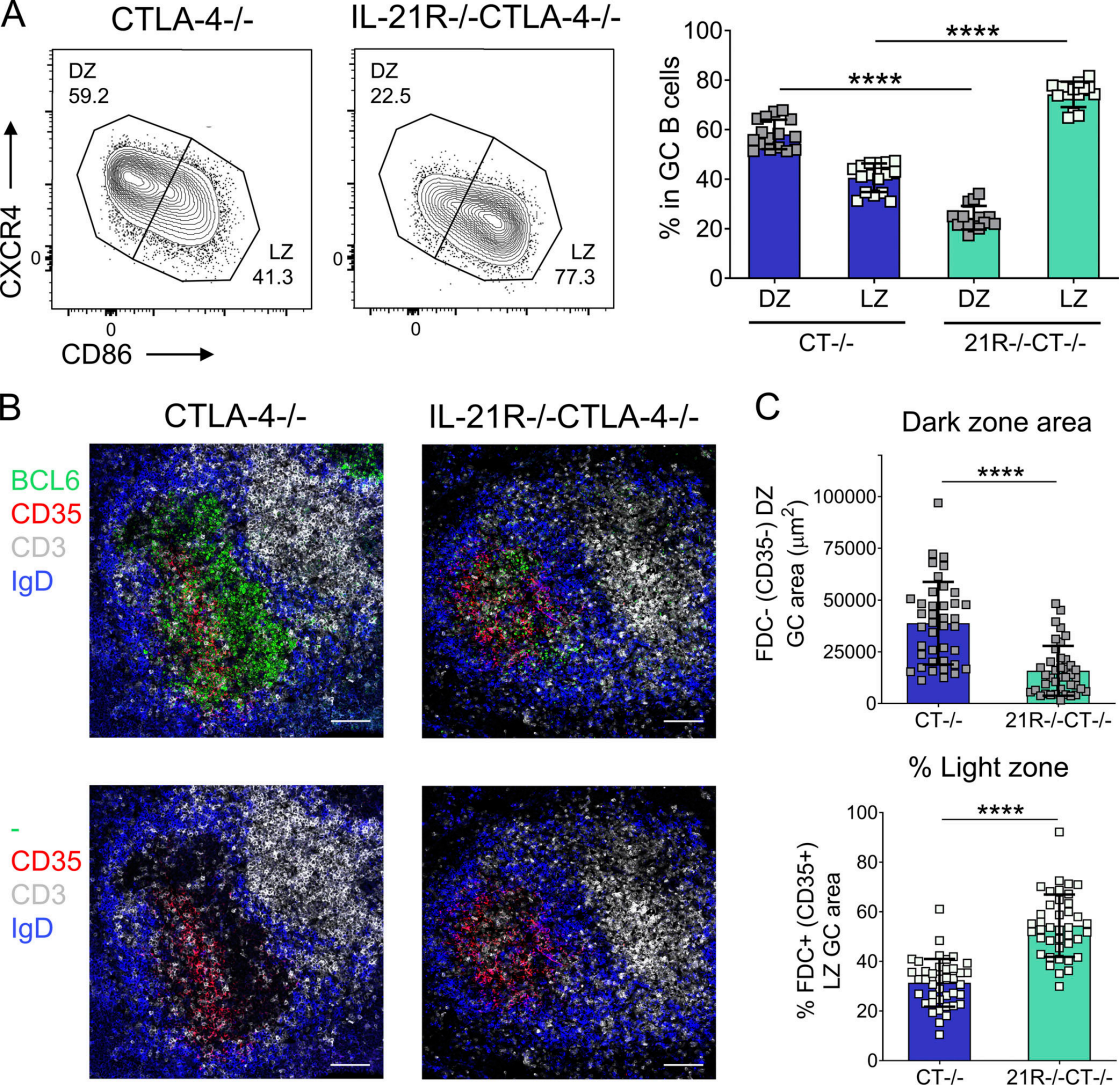
**IL-21R deficiency results in a profound GC dark zone defect in CTLA-4−/− mice. (A)** GC B cell distribution across dark zone (DZ; CXCR4^high^CD86^low^) and light zone (LZ; CXCR4^low^CD86^high^) compartments was analyzed in spleens from age-matched 16–21-d-old CTLA-4−/− and IL-21R−/−CTLA-4−/− mice. Representative flow cytometry plots (left) and collated data (right) showing frequencies of dark zone and light zone cells in the GC B cell compartment (CD19+CD138−BCL6+). Data are collated from seven independent experiments; *n* = 14–16; Kruskal–Wallis test. **(B and C)** GC stromal compartments were analyzed in spleen sections from 18–21-d-old CTLA-4−/− and IL-21R−/−CTLA-4−/− mice. FDC in GC light zone were identified by their CD35 expression. Spleen sections were stained for BCL6 (green), CD35 (red), CD3 (white), and IgD (blue). **(B)** Representative confocal images of splenic GC from 21-d-old CTLA-4−/− and IL-21R−/−CTLA-4−/− mice; scale bar 100 µm. **(C)** Collated data for dark zone absolute area (CD35−, top) and the percentage of GC area occupied by light zone stroma (CD35+, bottom) in splenic GC (10–11 GC/mouse; each point represents a GC). Data are collated from four independent experiments; *n* = 5; Mann–Whitney U test. Mean ± SD are shown; ****, P < 0.0001.

Previous reports have shown that IL-21 can downregulate CXCR4 and upregulate CD86 expression in activated murine B cells (Yoshida et al., 2011; Attridge et al., 2014). Since low CXCR4 and high CD86 expression are associated with a light zone GC B cell phenotype, we hypothesized that, in addition to their overall reduced GC B cell compartment, IL-21R−/−CTLA-4−/− mice may exhibit an impaired light zone GC B cell population. However, our analysis revealed the exact opposite (Fig. 2). While GC B cells in CTLA-4−/− mice exhibited dark zone (CXCR4^high^CD86^low^) and light zone (CXCR4^low^CD86^high^) phenotypes at an ∼60 to 40 ratio, which is consistent with dark zone GC B cells dominating GC in wild-type mice and in humans (Victora et al., 2012), in IL-21R−/−CTLA-4−/− mice, light zone GC B cells accounted for over 70% of the total GC B cell population (Fig. 2 A). Furthermore, while the absolute numbers of both splenic GC B cell populations were significantly reduced in IL-21R−/−CTLA-4−/− mice when compared with the CTLA-4−/− group, there was a more pronounced reduction in GC B cells with a dark zone phenotype (Fig. S1 D). Light zone skewing of GC B cells in IL-21R−/−CTLA-4−/− mice was further confirmed using CD83 and CD23 as additional markers of a light zone phenotype (data not shown). A greater requirement for IL-21R signals in GC dark zone development did not reflect elevated expression of IL-21R in dark zone GC B cells; instead, IL-21R expression in CTLA-4−/− mice was higher in light zone GC B cells (Fig. S1 E) consistent with the delivery of T cell–derived IL-21 in this location and in line with previous work in CTLA-4–sufficient mice (Zhang et al., 2018).

Light zone and dark zone GC B cell localization in situ is underpinned by the differentiation of chemokine-producing stromal cells. A subset of dark zone stromal cells produces CXCL12, which attracts CXCR4-expressing dark zone GC B cells, while the light zone compartment contains CD35^+^ follicular dendritic cells (FDC) that produce CXCL13 and aid localization of the CXCR5^+^CXCR4^−^ light zone GC B cells (Allen et al., 2004; Bannard et al., 2013). We, therefore, explored whether the perturbed GC B cell polarization in IL-21R–deficient CTLA-4−/− animals was associated with alterations in the GC stromal compartment. Analysis of spleen sections by confocal microscopy revealed profound changes in GC organization and stromal architecture in IL-21R−/−CTLA-4−/− mice. While GC in CTLA-4−/− mice were subdivided into distinct CD35^+^ light zone areas, distal to the T zone, and CD35^−^ dark zone areas, proximal to the T zone, in IL-21R−/−CTLA-4−/− mice, light zone–associated stroma dominated GC throughout (Fig. 2 B). Quantification revealed a significant decrease in GC dark zone area (Fig. 2 C, upper) but not light zone area (Fig. S1 F) in the absence of IL-21 signaling resulting in skewed GC light zone proportions (Fig. 2 C, lower). The altered histological appearance of GC in IL-21R−/−CTLA-4−/− mice was therefore consistent with the changes observed in GC B cell population composition by flow cytometry.

### IL-21 controls dark zone formation in immunization-induced GC

**Figure 3.**
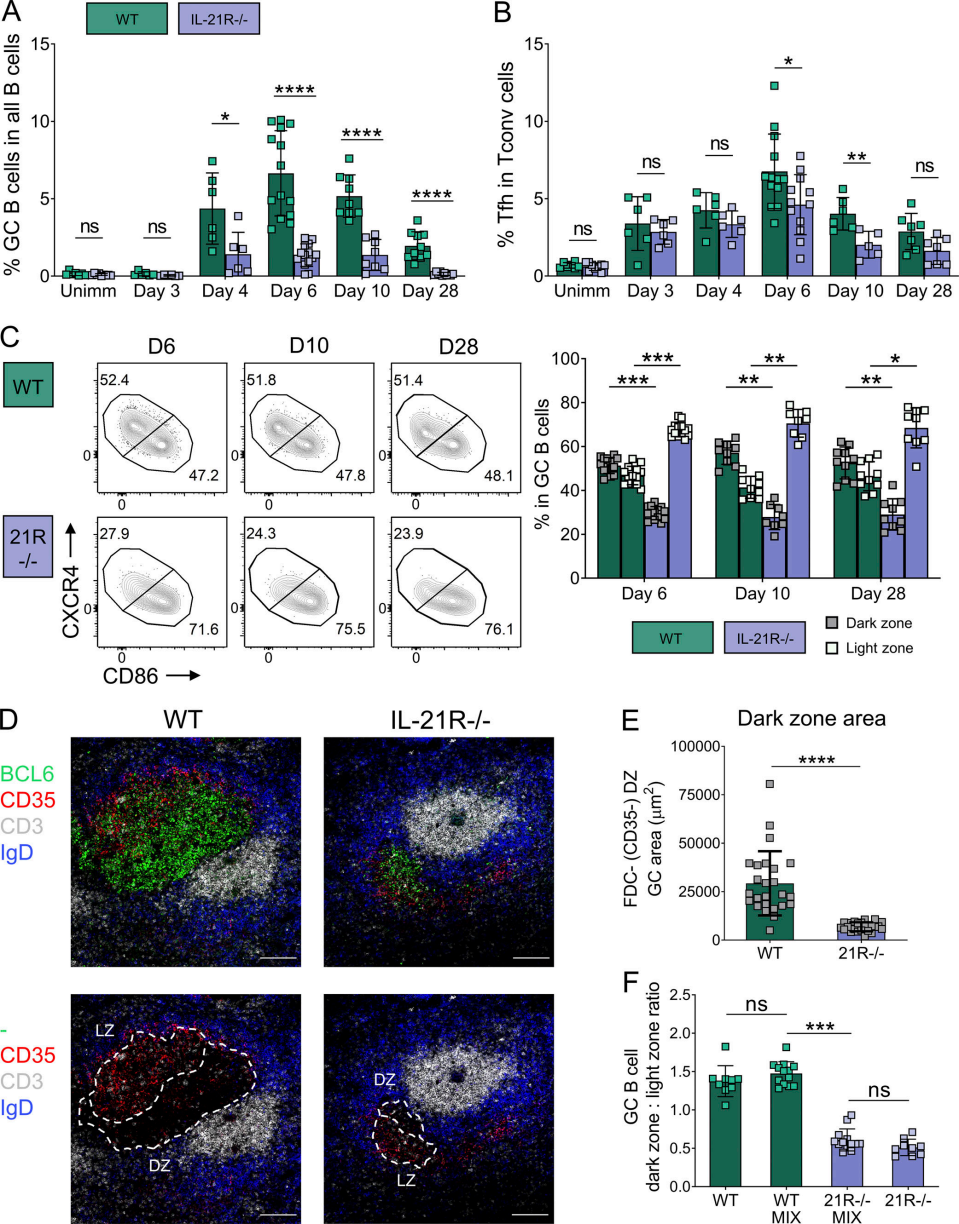
**GC B cells are skewed to a light zone phenotype in immunized IL-21R−/− mice.** Wild-type (WT) and IL-21R−/− BALB/c mice were immunized with SRBC and their splenic GC were analyzed 3, 4, 6, 10, or 28 d later along with unimmunized littermate controls (Unimm). **(A)** Collated data showing frequency of GC B cells (CD19+BCL6+) in all B cells (CD19+). **(B)** Collated data showing frequency of Tfh cells (CD3+CD4+FoxP3−BCL6+) in Tconv (CD3+CD4+FoxP3−). **(C)** GC B cell distribution across dark zone (CXCR4^high^CD86^low^) and light zone (CXCR4^low^CD86^high^) compartments. Representative flow cytometry plots (left) and collated data (right) showing frequencies of dark zone and light zone cells in the GC B cell compartment (CD19+BCL6+). Data are collated from seven independent experiments; *n* = 5–14; Kruskal–Wallis test. **(D and E)** GC compartments were analyzed in spleen sections from SRBC-immunized WT and IL-21R−/− mice 6 d after immunization. FDC in GC light zone were identified by their CD35 expression. Spleen sections were stained for BCL6 (green), CD35 (red), CD3 (white), and IgD (blue). **(D)** Representative confocal images of splenic GC from immunized WT and IL-21R−/− mice; scale bar 100 µm. **(E)** Collated data for dark zone (DZ) absolute area (CD35−) in splenic GC (7–9 GC/mouse; each point represents a GC). Data are collated from three independent experiments; *n* = 3; Mann–Whitney U test. **(F)** WT and IL-21R−/− mixed bone marrow chimeric mice (MIX) and recipients of just WT or IL-21R−/− bone marrow were immunized with SRBC and their spleens were analyzed 6 d later. Collated data showing the ratio of dark zone to light zone cells in the GC B cell compartment. Data are collated from two independent experiments; *n* = 9–13; Wilcoxon matched-pairs signed rank test. Mean ± SD are shown; ****, P < 0.0001; ***, P < 0.001; **, P < 0.01; *, P < 0.05; ns, not significant.

To investigate whether the GC dark zone defect observed in IL-21R–deficient CTLA-4−/− animals was secondary to the systemic immune dysregulation seen in the absence of CTLA-4, we compared GC from disease-free wild-type and IL-21R−/− animals responding to sheep red blood cell (SRBC) immunization. Immunization-induced GC were smaller in the absence of IL-21 signaling as previously reported (Zotos et al., 2010; Linterman et al., 2010; Zhang et al., 2018), and Tfh frequencies were also reduced at some time points (Fig. 3, A and B). Again, dark zone GC B cell frequencies and absolute numbers were significantly reduced in IL-21R−/− mice when compared with their wild-type counterparts, and this remained consistent across a range of timepoints (Fig. 3 C and Fig. S1 G). Consistent with the flow cytometry data, GC organization and stromal architecture in immunized IL-21R−/− mice were altered in situ. Although both dark zone and light zone areas in individual GC were reduced in immunized mice lacking IL-21R expression, the defect in the GC dark zone compartment was more pronounced, as evidenced by the dominance of CD35^+^ light zone stroma in IL-21R−/− GC (Fig. 3, D and E; and Fig. S1, H and I). To assess whether IL-21 promoted GC dark zone formation in a B cell–intrinsic manner, we constructed bone marrow chimeras containing a 50:50 mix of wild-type and IL-21R−/− hematopoietic cells. Following reconstitution, these animals were immunized with SRBC, and frequencies of wild-type and IL-21R−/− light zone and dark zone GC B cells were evaluated 6 d later. The IL-21R−/− GC B cell compartment remained markedly skewed toward a light zone phenotype even in the presence of wild-type immune cells and stroma, confirming B cell–intrinsic, IL-21–dependent regulation of GC B cell polarization (Fig. 3 F). Collectively, these data demonstrate that IL-21 is a key regulator of the GC B cell dark zone compartment in both chronic and immunization-induced GC.

### IL-21 does not decrease GC B cell apoptosis but promotes T cell–dependent selection of light zone GC B cells

**Figure S2.**
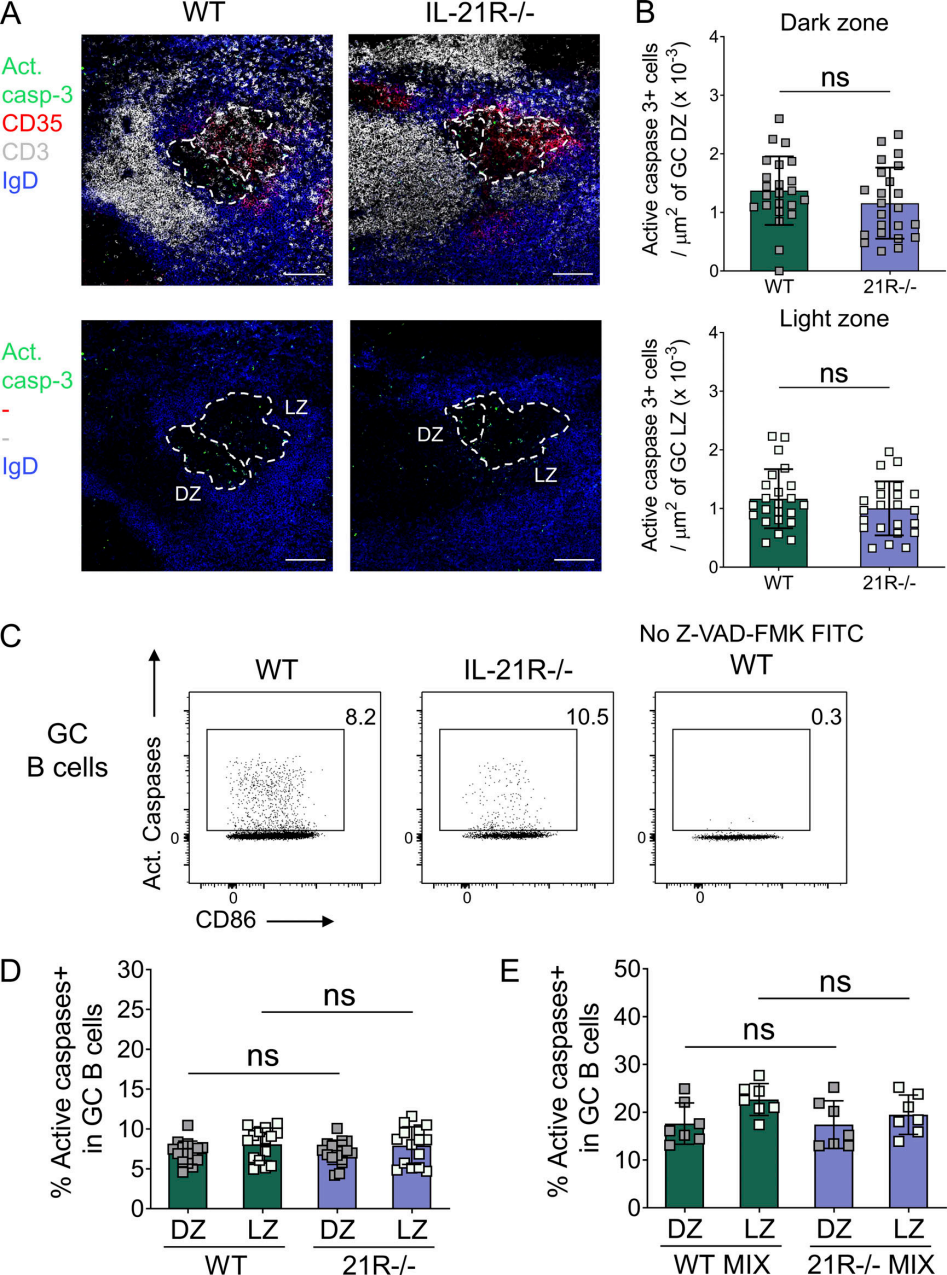
**GC B cells from SRBC-immunized wild-type and IL-21R−/− mice show comparable caspase activity.** **(A and B)** Density of apoptotic cells was analyzed in splenic GC from SRBC-immunized wild-type (WT) and IL-21R−/− mice 6 d after immunization. Spleen sections were stained for active caspase-3 (green), CD35 (red), CD3 (white), and IgD (blue). (A) Representative confocal images of splenic GC and (B) collated data comparing active caspase-3+ cell density in GC dark zone (DZ; CD35−; top) and light zone (LZ; CD35+; bottom). Scale bar 100 µm; 7–9 GC/mouse, each point represents a GC. Data are collated from two independent experiments; *n* = 3; Mann–Whitney U test. **(C and D)** Ex vivo, active caspases were detected using FITC-tagged active pan-caspase inhibitor Z-VAD-FMK. (C) Representative flow cytometry plots for caspase activity in GC B cells (live CD19+Fas+GL-7+) and (D) collated data for caspase activity in GC B cells subdivided into dark zone (CXCR4^high^CD86^low^) and light zone (CXCR4^low^CD86^high^) compartments. Data are collated from four independent experiments; *n* = 15; Kruskal–Wallis test. **(E)** WT and IL-21R−/− mixed bone marrow chimeric mice were immunized with SRBC and their spleens were analyzed 6 d later. Collated data for caspase activity in GC B cells subdivided into dark zone and light zone compartments. Data are collated from two independent experiments; *n* = 7; Wilcoxon matched-pairs signed rank test. Mean ± SD are shown; ns, not significant.

Apoptosis is prevalent within the GC, impacting up to 50% of GC B cells over a 6-h period (Mayer et al., 2017) and necessitating dedicated removal by specialized tangible body macrophages (Gurwicz et al., 2023). Dark zone loss in IL-21R–deficient mice was not attributable to increased cell death since we observed a comparable distribution of active caspase 3–expressing apoptotic cells in dark zone and light zone areas of splenic GC from immunized IL-21R–sufficient and IL-21R–deficient mice in situ (Fig. S2, A and B). Similarly, we found equivalent caspase activity in GC B cells from wild-type and IL-21R−/− mice when cells were analyzed ex vivo using a fluorescent irreversible pan-caspase inhibitor (“CaspGLOW” assay as described by Good-Jacobson et al., 2010; Fig. S2, C and D), and this was recapitulated in mixed bone marrow chimeric mice (Fig. S2 E). These findings are in line with previous data from IL-21R–deficient mice, although light and dark zones were not analyzed separately in these studies (Gonzalez et al., 2018; Dvorscek et al., 2022).

Formation of the GC dark zone involves seeding by selected light zone GC B cells, with T cell–derived signals being critical to permit affinity-based discrimination (Victora et al., 2010). This reflects the capacity of T cells to provide metabolic “refueling” to GC B cells in an affinity-dependent manner, instilling the biochemical pathways required for dark zone maintenance (Long et al., 2022). Since molecular players implicated in refueling include basic leucine zipper ATF-like transcription factor (BATF) and mammalian target of rapamycin complex 1 (mTORC1), both targets of IL-21 signaling (Inoue et al., 2017; Ersching et al., 2017; Xin et al., 2015; Kato and Perl, 2018), this raised the possibility of a role for IL-21 in this step. While the involvement of CD40 ligand (CD40L) signaling in Tfh cell–dependent light zone GC B cell selection has already been demonstrated (Inoue et al., 2017; Luo et al., 2018), the contribution of Tfh cell cytokines to this process is less well understood.

**Figure 4.**
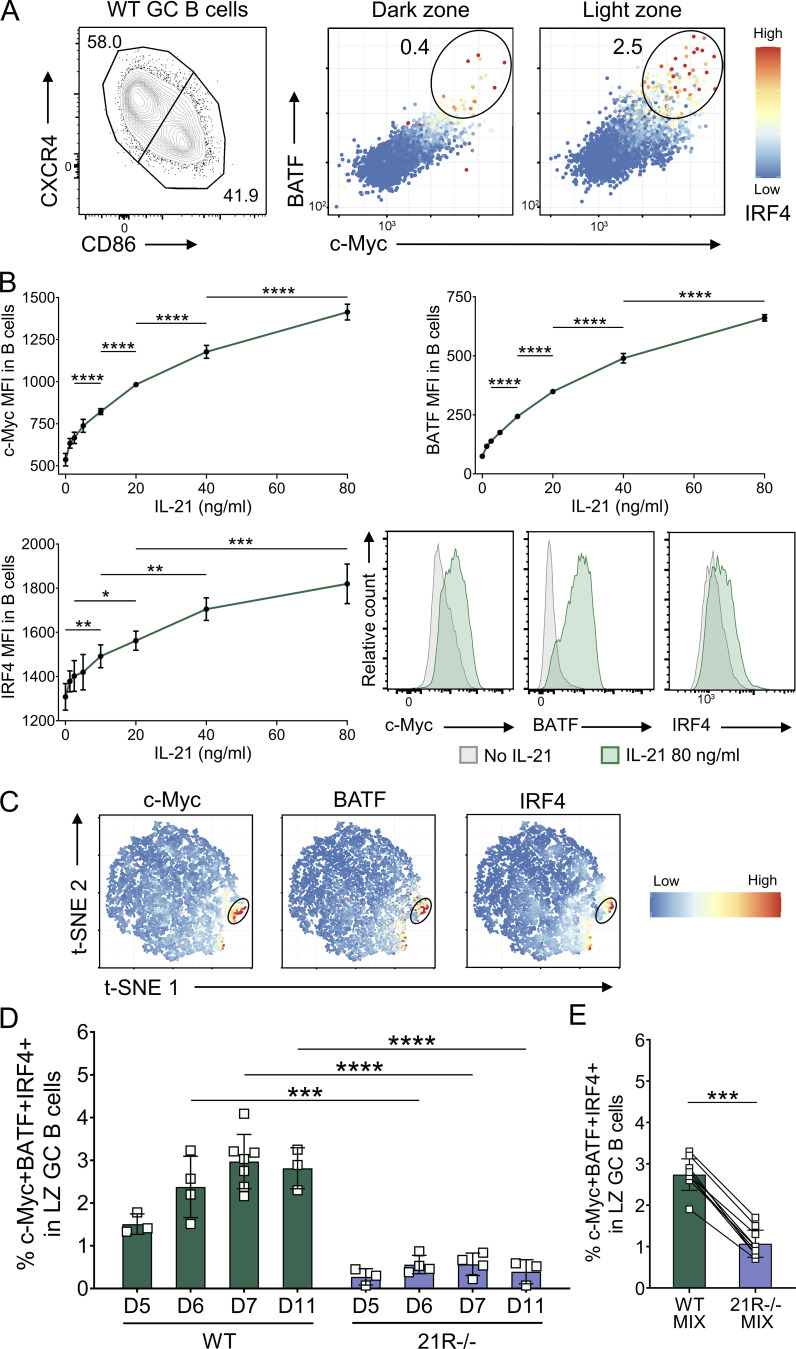
**IL-21 promotes light zone GC B cell selection. (A)** Representative flow cytometry plots showing c-Myc, BATF, and IRF4 expression in splenic dark zone (CXCR4^high^CD86^low^) and light zone (CXCR4^low^CD86^high^) GC B cells from SRBC-immunized wild-type (WT) mice 6 d after immunization; color scales define IRF4 expression levels in each cell. **(B)** CD19+ cells were isolated from spleens of 10-wk-old WT BALB/c mice using magnetic-activated cell sorting (MACS) positive selection. Cells were stimulated using 0–80 ng/ml of IL-21 and 20 μg/ml of anti-CD40 mAb for 24 h. Collated data and representative histograms for c-Myc, BATF, and IRF4 median fluorescence intensity (MFI). One-way ANOVA; *n* = 4. **(C)** Representative t-SNE plots showing expression of c-Myc, BATF, and IRF4 in concatenated WT and IL-21R−/− light zone GC B cells (6 d after immunization); color scales define protein expression levels in each cell. **(D)** Collated data for c-Myc+BATF+IRF4+ splenic light zone (LZ) GC B cell frequencies in WT and IL-21R−/− mice at 5, 6, 7, or 11 d after immunization. Data are collated from four independent experiments; *n* = 3–7; Mann–Whitney U test. **(E)** WT and IL-21R−/− mixed bone marrow chimeric mice were immunized i.p. with 2 × 10^9^ SRBC and spleens were analyzed 6 d later. Collated data for c-Myc+BATF+IRF4+ cell frequencies in splenic WT and IL-21R−/− light zone GC B cells in mixed bone marrow chimeric mice. Data are collated from two independent experiments; *n* = 10; Wilcoxon matched-pairs signed rank test. Mean ± SD are shown; ****, P < 0.0001; ***, P < 0.001; **, P < 0.01; *, P < 0.05.

**Figure S3.**
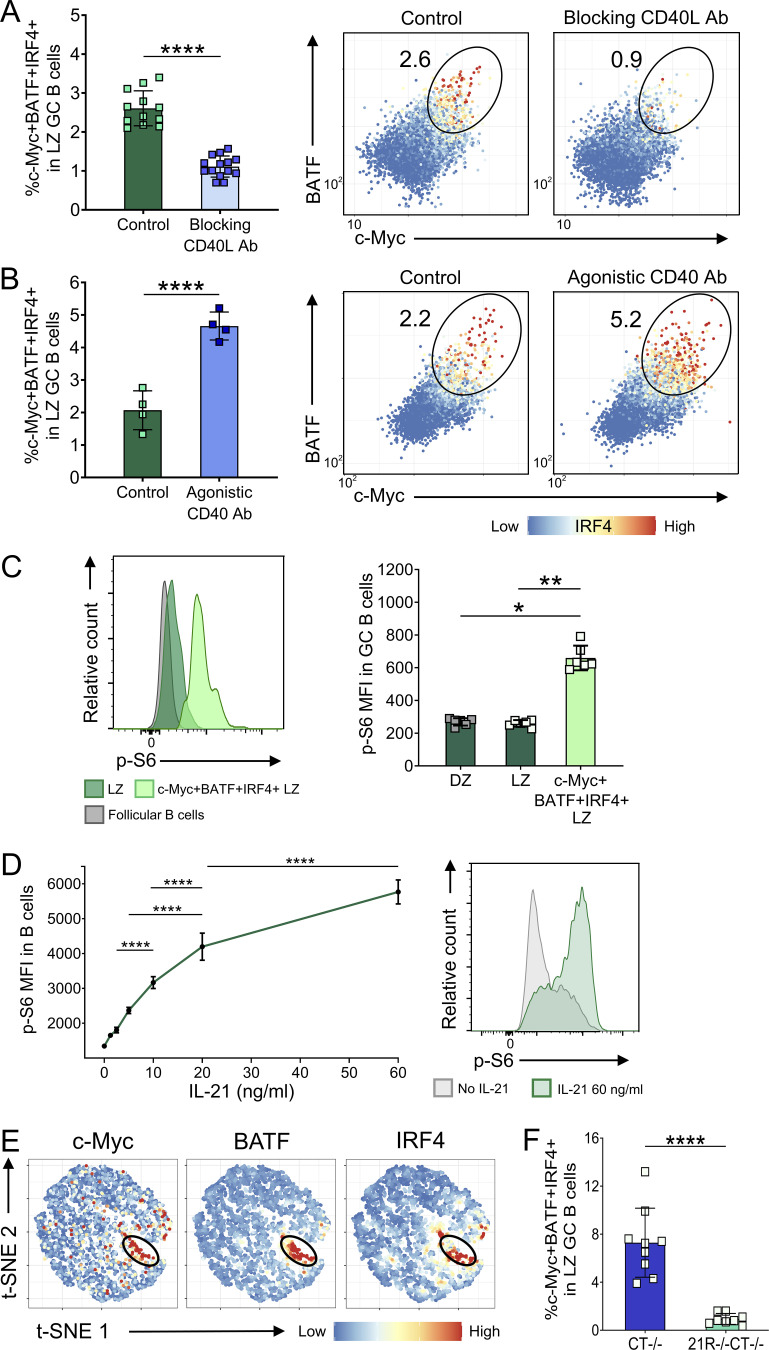
**IL-21 regulates light zone GC B cell selection. (A)** Wild-type (WT) mice were immunized with SRBC and either treated with blocking anti-CD40L antibody (MR1; 200 μg/i.p. injection on days 5 and 6 after immunization) or received no further treatment (Control). Collated data (left) and representative flow cytometry plots (right) for c-Myc+BATF+IRF4+ splenic light zone (LZ; CXCR4^low^CD86^high^) GC B cell (CD19+BCL6+) frequencies 7 d after immunization; color scales define IRF4 expression levels in each cell. **(B)** WT mice were immunized with SRBC and either treated with agonistic CD40 antibody (FGK4.5; 50 μg i.p on day 5 after immunization) or received no further treatment (Control). Collated data (left) and representative flow cytometry plots (right) for c-Myc+BATF+IRF4+ splenic light zone GC B cell frequencies 6 d after immunization; color scales define IRF4 expression levels in each cell. **(C)** p-S6 expression was analyzed in splenic GC from SRBC-immunized WT mice 6 d after immunization. Representative histograms showing p-S6 expression mean fluorescence intensity (MFI) in c-Myc+BATF+IRF4+ and c-Myc−BATF−IRF4− light zone GC B cells compared to follicular B cells (CD19+IgD+BCL6−; left). Collated data for p-S6 expression in c-Myc+BATF+IRF4+ and c-Myc−BATF−IRF4− light zone and all dark zone (DZ; CXCR4^high^CD86^low^) GC B cells (right). Data are collated from two to four independent experiments; *n* = 4–14; Mann–Whitney U test or Kruskal–Wallis test. **(D)** CD19+ cells were isolated from spleens of 10-wk-old WT BALB/c mice using MACS positive selection. Cells were stimulated using 0–60 ng/ml of IL-21 and 20 μg/ml of anti-CD40 mAb for 24 h. Collated data and representative histograms for p-S6 median fluorescence intensity. One-way ANOVA; *n* = 4. **(E)** Representative t-SNE plots showing expression of c-Myc, BATF, and IRF4 in concatenated CTLA-4−/− and IL-21R−/−CTLA-4−/− light zone GC B cells; color scales define protein expression levels in each cell. **(F)** Collated data for c-Myc+BATF+IRF4+ splenic light zone GC B cell frequencies in 17–21-d-old CTLA-4−/− and IL-21R−/−CTLA-4−/− mice. Data are collated from five independent experiments; *n* = 8–9; Mann–Whitney U test. Mean ± SD are shown; ****, P < 0.0001; **, P < 0.01; *, P < 0.05.

Detailed analysis by several groups has shed light on the phenotype of T cell–selected GC B cells, beginning with the discovery that a small subset of GC B cells expressed c-Myc; these cells had a light zone GC phenotype, expressed interferon regulatory factor 4 (IRF4), and were interspersed among FDC (Calado et al., 2012; Dominguez-Sola et al., 2012). Antigen targeting with Dec205 antibodies revealed that c-Myc+ light zone GC B cells formed in a T cell–dependent manner (Dominguez-Sola et al., 2012) with c-Myc being induced in proportion to antigen capture (Finkin et al., 2019). Finally, BATF was revealed as a marker of T cell–dependent selection, being induced in GC B cells in an MHC class II and CD40L-dependent manner (Inoue et al., 2017). Informed by these pioneering studies, we tested whether flow cytometric staining for c-Myc, IRF4, and BATF could be used to identify selected light zone GC B cells. We were able to identify a distinct GC B cell population that co-expressed these three markers and was present among light zone but not dark zone GC B cells following SRBC immunization (Fig. 4 A). This population was significantly decreased if T cell help was curtailed via injection of blocking anti-CD40L antibody, and conversely, it increased following treatment with agonistic anti-CD40 antibody (Fig. S3, A and B). T cell help is known to drive activation of the mTORC1 pathway in light zone GC B cells and selected cells express higher levels of phosphorylated ribosomal protein S6 (p-S6; Ersching et al., 2017). The population of c-Myc+BATF+IRF4+ cells we identified expressed significantly higher p-S6 than other light zone GC B cells (Fig. S3 C) consistent with T cell–dependent selection. IL-21 increased the expression of c-Myc, BATF, and IRF4 in a dose-dependent manner in splenic B cells cultured in vitro (Fig. 4 B), and IL-21 also increased p-S6 in line with its ability to promote AKT phosphorylation (Dvorscek et al., 2022; Fig. S3 D). While the findings from our assays suggested that IL-21 had the capacity to activate selection-associated cellular pathways in B cells in vitro, to investigate if IL-21 indeed promoted light zone GC B cell selection for dark zone recycling in vivo, we examined the c-Myc+BATF+IRF4+ B cell population within the GC of wild-type and IL-21R−/− mice. Strikingly, when frequencies of these cells expressing Tfh-dependent selection-associated transcription factors were assessed, they were found to be present and follow GC kinetics in IL-21R–sufficient animals while being almost undetectable in IL-21R–deficient mice (Fig. 4, C and D). To assess whether IL-21 could promote the formation of the c-Myc+BATF+IRF4+ light zone GC B cell population in a cell-intrinsic manner, we compared their frequencies in SRBC immunized wild-type and IL-21R−/− mixed bone marrow chimeras that were constructed as described above. While cells co-expressing c-Myc, BATF, and IRF4 could be detected among wild-type light zone GC B cells, this cell subset was greatly reduced amongst their IL-21R–deficient counterparts (Fig. 4 E). The c-Myc, BATF, and IRF4 co-expressing light zone GC B cell population could also be readily identified in chronic GC in CTLA-4–deficient mice, where they were present at even higher frequencies than in immunized CTLA-4–sufficient animals, and again were virtually abolished in the absence of IL-21R (Fig. S3, E and F). Taken together, these data indicate a role for IL-21 in the T cell–dependent selection and metabolic refueling of light zone GC B cells, likely contributing to the dark zone defect observed in IL-21R deficiency.

### IL-21 upregulates cyclin D3 expression in GC B cells

**Figure 5.**
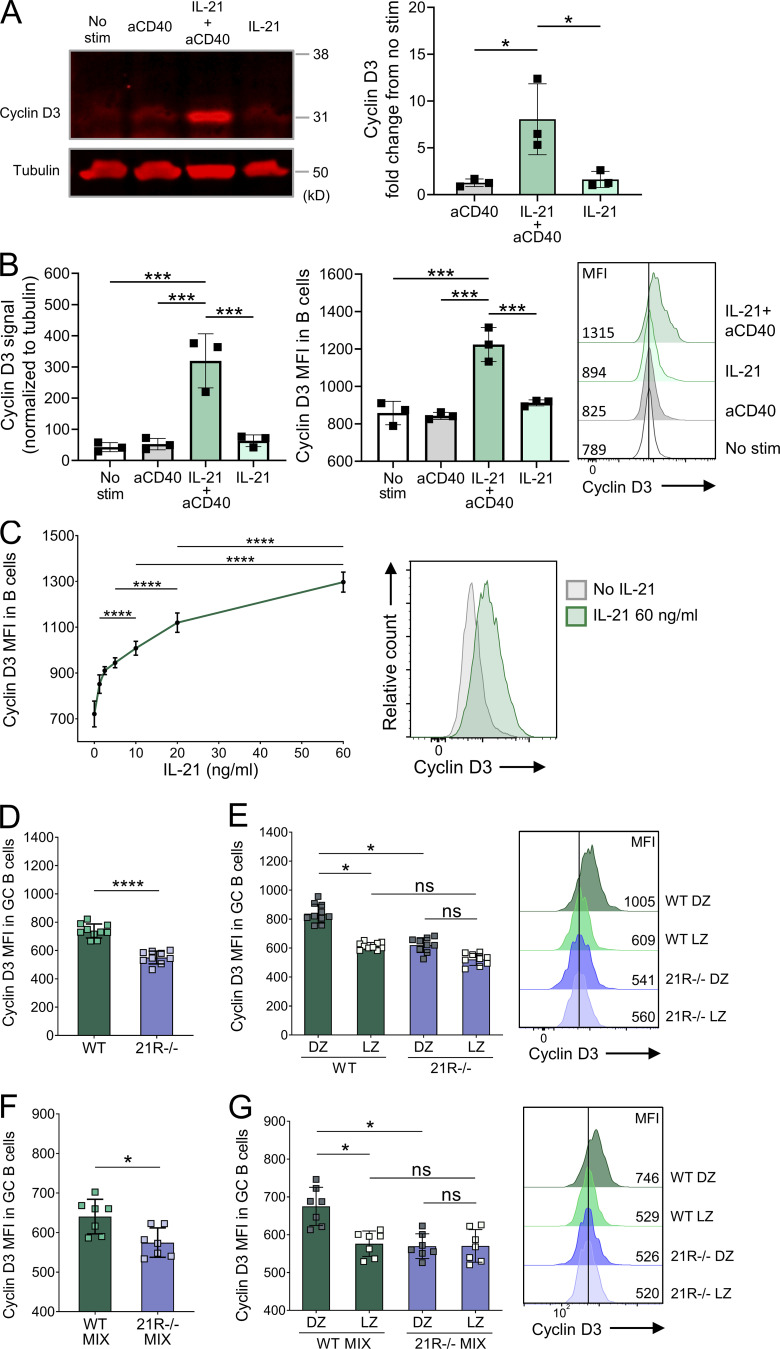
**IL-21 promotes cyclin D3 expression in GC B cells.** CD19+ cells were isolated from spleens of 7–15-wk-old wild-type (WT) BALB/c mice using MACS positive selection. Cells were treated with 80 ng/ml of IL-21 and/or 20 μg/ml of anti-CD40 mAb as indicated for 24 h, and cell lysates were examined by Western blotting and flow cytometry. **(A)** Representative Western blots for cyclin D3 and tubulin and collated data showing cyclin D3 expression fold change from untreated cells normalized to tubulin. **(B)** Collated Western blot data showing cyclin D3 expression normalized to tubulin (left), and collated data and representative histograms (right) showing cyclin D3 expression mean fluorescence intensity (MFI) measured by flow cytometry. One-way ANOVA; *n* = 3. **(C)** CD19+ cells were treated with 0–60 ng/ml of IL-21 and 20 μg/ml of anti-CD40 mAb for 24 h and examined by flow cytometry. Collated data and representative histograms showing cyclin D3 mean fluorescence intensity. One-way ANOVA; *n* = 3–4. Cyclin D3 expression was analyzed in splenic GC from SRBC-immunized WT and IL-21R−/− mice 6 d after immunization. **(D)** Collated data showing cyclin D3 expression mean fluorescence intensity in GC B cells (CD19+BCL6+). **(E)** Collated data and representative histograms showing cyclin D3 expression mean fluorescence intensity in dark zone (DZ; CXCR4^high^CD86^low^) and light zone (LZ; CXCR4^low^CD86^high^) GC B cells. Data are collated from three independent experiments; *n* = 9–10; Kruskal–Wallis or Mann–Whitney U test. WT and IL-21R−/− mixed bone marrow chimeric mice were immunized with SRBC and their spleens were analyzed 6 d later. **(F)** Collated data showing cyclin D3 expression mean fluorescence intensity in WT and IL-21R−/− GC B cells. **(G)** Collated data and representative histograms showing cyclin D3 expression mean fluorescence intensity in WT and IL-21R−/− dark zone and light zone GC B cells. Data are collated from two independent experiments; *n* = 7; Kruskal–Wallis or Mann–Whitney U test. Mean ± SD are shown; ****, P < 0.0001; ***, P < 0.001; *, P < 0.05; ns, not significant. Source data are available for this figure: SourceData F5.

**Figure S4.**
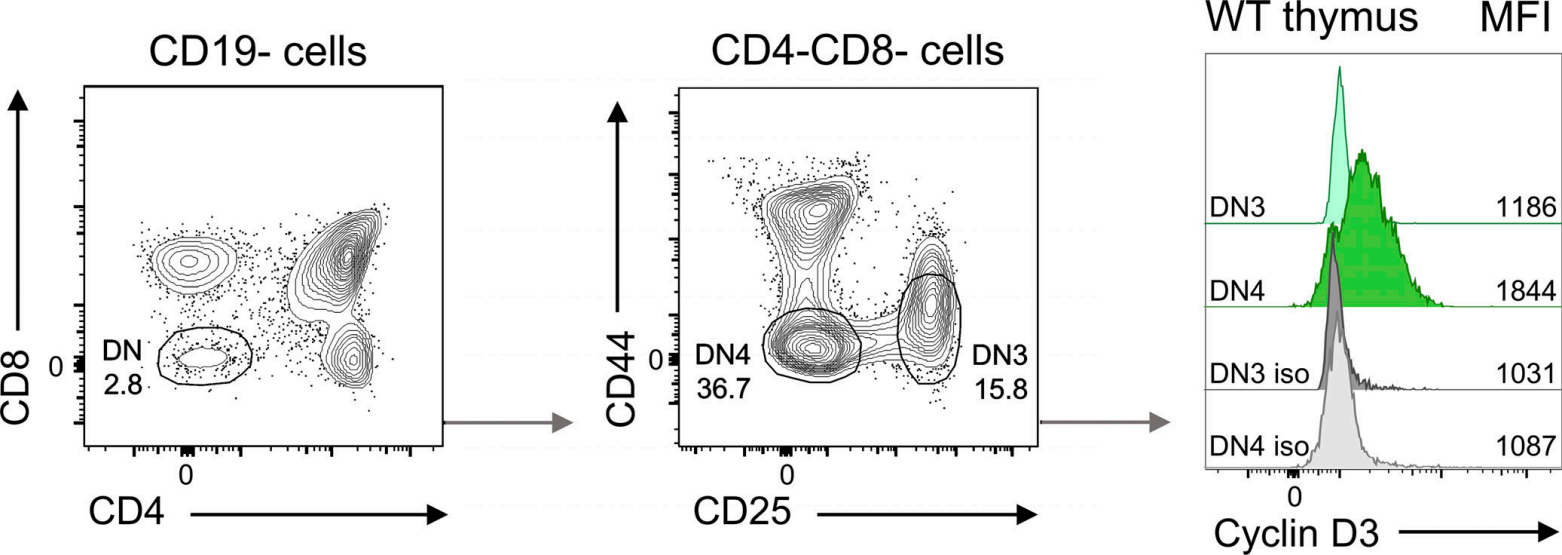
**Validation of cyclin D3 flow cytometry staining using wild-type thymocytes.** Thymocytes from wild-type (WT) BALB/c mice were stained with the DCS-22 anti-cyclin D3 antibody or isotype control (iso). Representative flow cytometry plots showing thymocyte subset gating strategy and cyclin D3 expression mean fluorescence intensity (MFI) in double-negative 3 (DN3) and double-negative 4 (DN4) thymocytes.

**Figure S5.**
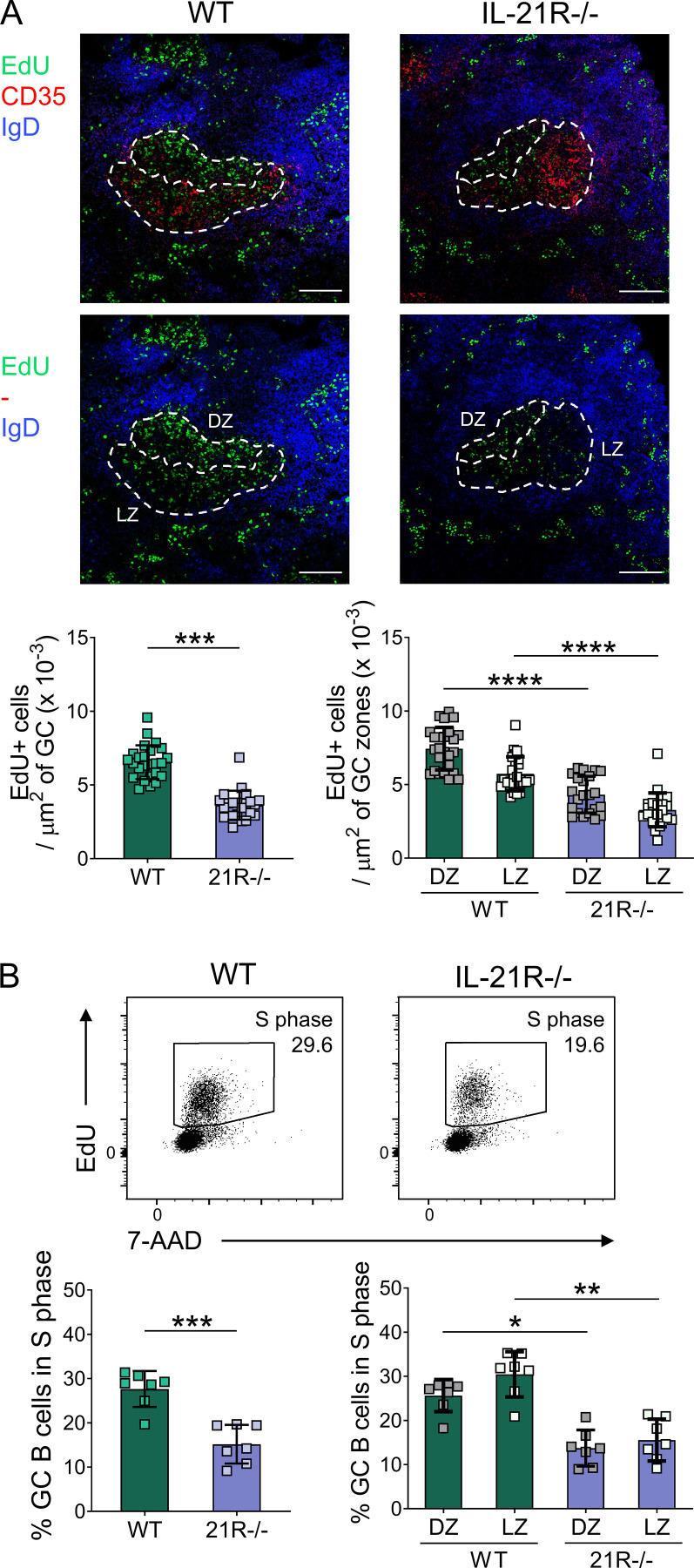
**IL-21 promotes proliferation of GC B cells.** Proliferating cells were analyzed in splenic GC from SRBC-immunized wild-type (WT) and IL-21R−/− mice 6 d after immunization. Mice received i.p. injections of EdU 30 min before tissue harvest. **(A)** Representative confocal images (top) of splenic GC and collated data (bottom) comparing EdU+ cell density in total GC and in GC dark zone (DZ; CD35^−^) and GC light zone (LZ; CD35^+^) areas from immunized WT and IL-21R−/− mice (scale bar 100 µm; 7–9 GC/mouse, each point represents a GC). Spleen sections were stained for EdU (green), CD35 (red), and IgD (blue). Data are collated from two independent experiments; *n* = 3; Mann–Whitney U test or Kruskal–Wallis test. **(B)** Representative flow cytometry plots (top) showing splenic GC B cells (CD19+Fas+GL-7+) in S phase (EdU+) and collated data (bottom) for total GC B cells and dark zone (CXCR4^high^CD86^low^) and light zone (CXCR4^low^CD86^high^) GC B cells in S phase. Data are collated from two independent experiments; *n* = 7; Mann–Whitney U test or Kruskal–Wallis test. Mean ± SD are shown; ****, P < 0.0001; ***, P < 0.001**, P < 0.01; *, P < 0.05.

The dark zone is the focus of GC B cell proliferation, while the duration of proliferation is indexed via a cellular “timer” (Bannard et al., 2013) to the amount of antigen that GC B cells capture and present to Tfh (Gitlin et al., 2014); ongoing dark zone proliferation occurs independently of further T cell help and has been termed “inertial” (Pae et al., 2021). Recent studies have demonstrated that inertial dark zone GC B cell proliferation is controlled in a dose-dependent manner by the cell cycle regulator cyclin D3 (Ramezani-Rad et al., 2020; Pae et al., 2021). Strikingly, mice deficient in cyclin D3 were shown to exhibit a profound loss of the GC dark zone compartment and a light zone skewing phenotype highly reminiscent of that seen in our IL-21R–deficient systems (Pae et al., 2021; Ramezani-Rad et al., 2020). We, therefore, questioned whether IL-21 controlled GC dark zone homeostasis by regulating the expression of cyclin D3. Western blot analysis in splenic CD19^+^ B cells revealed that IL-21 greatly upregulated cyclin D3 in the presence of CD40 engagement (Fig. 5 A), consistent with the reported synergy between CD40 and IL-21 in activating B cell metabolic and biosynthetic pathways in vitro (Luo et al., 2023; Chen et al., 2023). To study cyclin D3 protein expression, we optimized our flow cytometry staining using murine thymocytes in which cyclin D3 has been shown to be strongly induced at the double-negative 4 stage (Sicinska et al., 2003; Fig. S4). Further comparison with Western blotting confirmed that flow cytometry could also be used to assess cellular cyclin D3 levels in murine B cells (Fig. 5 B). Titration experiments demonstrated that rather than acting as an on/off switch for cyclin D3 upregulation, IL-21 modulated cyclin D3 levels in a dose-dependent manner (Fig. 5 C). To test whether IL-21 could also increase cyclin D3 expression within the GC B cell compartment in vivo, GC were induced by SRBC immunization. Analysis of cyclin D3 levels in GC B cells from wild-type and IL-21R−/− animals revealed a significant decrease in cyclin D3 expression in the absence of IL-21 signaling (Fig. 5 D). Past reports have demonstrated higher cyclin D3 in the GC dark zone (Pae et al., 2021); however, the reduced cyclin D3 levels in IL-21R−/− GC B cells were not simply a consequence of their smaller dark zone compartment since dark zone GC B cells from IL-21R–deficient mice expressed significantly lower levels of cyclin D3 compared to their wild-type counterparts even when each GC B cell subset was studied separately (Fig. 5 E). The reduction in GC B cell cyclin D3 expression in the absence of IL-21 signals was also observed in mixed bone marrow chimeric mice confirming that the effect was B cell intrinsic (Fig. 5, F and G). Consistent with the role of cyclin D3 in promoting cell proliferation, 5-ethynyl-2′-deoxyuridine (EdU) incorporation was significantly reduced in GC B cells from IL-21R–deficient mice in line with published literature (Zotos et al., 2021; Zotos et al., 2010; Gonzalez et al., 2018; Fig. S5, A and B). Collectively these data link GC zonal skewing in IL-21R deficiency to a defect in cyclin D3 upregulation and dark zone inertial proliferation.

### IL-21 modulates B cell Foxo1 expression and localization

**Figure 6.**
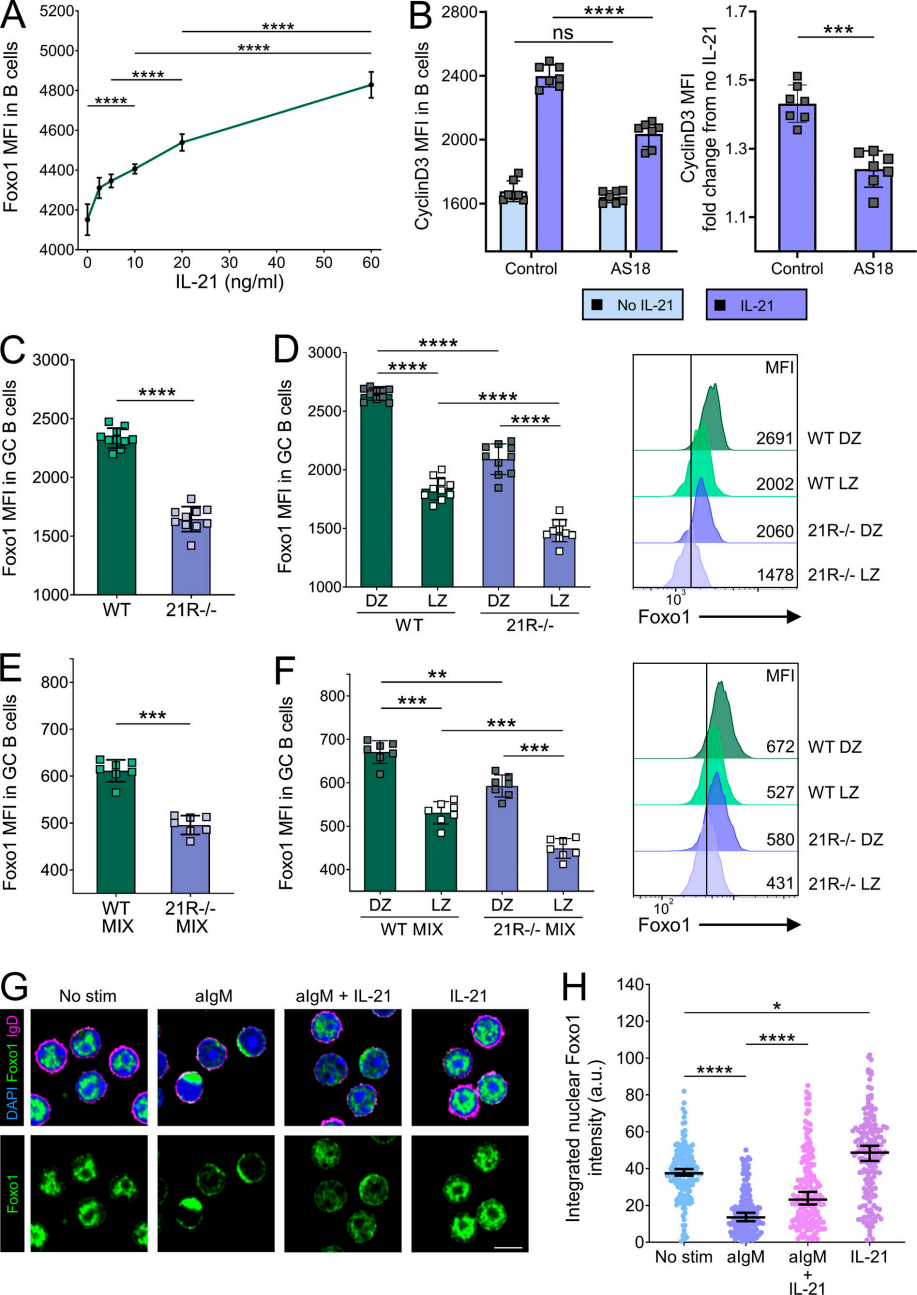
**IL-21 promotes Foxo1 expression in GC B cells.** CD19+ cells were isolated from spleens of 10-wk-old wild-type (WT) BALB/c mice using MACS positive selection. **(A)** Cells were stimulated using 0–60 ng/ml of IL-21 and 20 μg/ml of anti-CD40 mAb for 24 h. Collated data showing Foxo1 mean fluorescence intensity (MFI). Cells were stimulated with 20 μg/ml of anti-CD40 mAb with or without 80 ng/ml of IL-21 in the presence or absence of 2 μM of Foxo1 inhibitor AS1842856 (AS18) for 16 h. One-way ANOVA; *n* = 4. **(B)** Collated data showing cyclin D3 mean fluorescence intensity (left) and collated data showing cyclin D3 expression fold change from cells incubated in the absence of IL-21 (right). Data are collated from two independent experiments; *n* = 7; Kruskal–Wallis or Mann–Whitney U test. Foxo1 expression was analyzed in splenic GC from SRBC-immunized WT and IL-21R−/− mice 6 d after immunization. **(C)** Collated data showing Foxo1 expression mean fluorescence intensity in GC B cells (CD19+BCL6+). **(D)** Collated data and representative histograms showing Foxo1 expression mean fluorescence intensity in dark zone (DZ; CXCR4^high^CD86^low^) and light zone (LZ; CXCR4^low^CD86^high^) GC B cells. Data are collated from three independent experiments; *n* = 10; Kruskal–Wallis or Mann–Whitney U test. WT and IL-21R−/− mixed bone marrow chimeric mice were immunized with SRBC and their spleens were analyzed 6 d later. **(E)** Collated data showing Foxo1 expression mean fluorescence intensity in WT and IL-21R−/− GC B cells. **(F)** Collated data and representative histograms showing Foxo1 expression mean fluorescence intensity in WT and IL-21R−/− dark zone and light zone GC B cells. Data are collated from two independent experiments; *n* = 7; Kruskal–Wallis or Mann–Whitney U test. Mean ± SD are shown. CD19+ splenocytes (MACS separated) were left unstimulated or treated with 10 μg/ml of anti-IgM Ab for 10 min followed by either 30 min stimulation with 80 ng/ml of IL-21 or no further treatment. The localization of Foxo1 was examined by confocal microscopy. **(G)** Representative confocal microscopy images of CD19+ splenocytes from all treatment groups. Cells were stained with DAPI (blue), Foxo1 (green), and IgD (magenta); scale bar 5 µm. **(H)** Collated data showing nuclear Foxo1 intensity across treatment groups. Kruskal–Wallis test; *n* = 170–182 (each point represents a cell). Median ± 95% confidence interval are shown; ****, P < 0.0001; ***, P < 0.001; **, P < 0.01; *, P < 0.05; ns, not significant.

Cyclin D3 gene transcription is directly regulated by the transcription factor Foxo1 (Zhang et al., 2016; Ketzer et al., 2022; Wang et al., 2018), and Foxo1-deficient GC B cells also exhibit light zone skewing (Dominguez-Sola et al., 2015; Sander et al., 2015). We therefore examined the role of Foxo1 in IL-21–mediated cyclin D3 upregulation. We found that IL-21 was capable of dose-dependent upregulation of Foxo1 in splenic B cells in vitro (Fig. 6 A). Furthermore, we demonstrated that pharmacological inhibition of Foxo1 reduced the extent of IL-21–dependent cyclin D3 upregulation (Fig. 6 B). The incomplete inhibition of cyclin D3 upregulation observed suggests that Foxo1 is one of several pathways linking IL-21 to cyclin D3. To examine whether IL-21 was also able to regulate Foxo1 levels in GC B cells, we analyzed splenocytes from SRBC-immunized wild-type and IL-21R–deficient animals. GC B cells from IL-21R−/− mice exhibited lower levels of Foxo1 than their wild-type counterparts (Fig. 6, C and D), and Foxo1 analysis in GC B cells from bone marrow chimeric mice confirmed that this phenotype was B cell intrinsic (Fig. 6, E and F), suggesting a role for IL-21 in modulating GC B cell Foxo1 expression. Reduced levels of Foxo1 may therefore contribute to the GC dark zone defect in IL-21R−/− mice.

The nuclear localization of Foxo1 is important for its ability to instruct the GC dark zone transcriptional program (Dominguez-Sola et al., 2015; Sander et al., 2015). B cell receptor ligation is known to lead to Foxo1 phosphorylation and its rapid displacement from the nucleus to the cytoplasm (Luo et al., 2018), while data from CD4 T cells suggest STAT3-activating cytokines can facilitate nuclear localization of Foxo1 (Oh et al., 2012). We therefore questioned whether IL-21 might aid relocation of Foxo1 back to the nucleus in B cells, permitting transcriptional regulation of cyclin D3 and other dark zone–associated target genes. Confocal microscopy analysis of splenic B cells demonstrated that anti-IgM treatment efficiently relocated Foxo1 from the nucleus to the cytoplasm; however, exposure to IL-21 significantly counteracted this effect (Fig. 6, G and H). Taken together, these data suggest that one way in which IL-21 can regulate cyclin D3 expression and the dark zone program is by modulating the expression level and localization of Foxo1.

## Discussion

The generation of optimal humoral responses relies on the ability of B cells to integrate cues from a complex network of cellular and molecular interactions that shape B cell fate decisions across GC spatio-temporal axes (Victora and Nussenzweig, 2022). Tfh cell–derived IL-21 represents one such cue; however, its distinct contributions relative to those directed by CD40 engagement have not been clear. Here, we identify key roles for the Tfh cytokine IL-21 in B cell selection in the GC light zone and in directing cyclin D3–dependent inertial cell cycling in the GC dark zone.

Our observation that GC B cells are skewed to a light zone phenotype in the absence of IL-21 signaling is consistent with data obtained from adoptively transferred IL-21R−/− B cells responding to 4-hydroxy-3-nitrophenyl acetyl (NP)-OVA in alum (Gonzalez et al., 2018), as well as IL-21R−/− C57Bl/6 mice immunized with NP-KLH in alum (Zotos et al., 2021). In the latter study, transcriptional analysis of isolated NP-binding GC B cells confirmed enrichment of light zone signature genes in the absence of IL-21R but did not identify alterations in genes associated with light zone GC B cell selection. Here, we identified a population of B cells co-expressing c-Myc, BATF, and IRF4 within the GC light zone and showed that IL-21R deficiency led to a marked reduction of this population in vivo. Of note, the low frequency of c-Myc+BATF+IRF4+ light zone GC B cells detected by flow cytometry explains why these would have been missed in earlier bulk mRNA sequencing studies. It is known that B cell receptor and CD40 signaling synergize to induce c-Myc expression in GC B cells (Luo et al., 2018; Yam-Puc et al., 2021), and our data together with recent reports on combined B cell signal outcomes (Luo et al., 2023; Chen et al., 2023; Di Pietro et al., 2022) now pinpoint IL-21 as a key contributor to the development of this phenotype.

Cyclin D3 was previously shown to be important for GC maturation in vivo despite being largely dispensable for B cell proliferation and class switching in vitro (Peled et al., 2010). This was recently shown to reflect a critical role for cyclin D3 in GC dark zone inertial cell cycling (Pae et al., 2021). D cyclins are distinct from cyclins whose expression oscillates with the cell cycle phases; instead, they act as growth factor sensors that are attuned to mitogenic inputs, with their transcription, assembly, nuclear transport, and turnover all subject to regulation by external cues (Sherr and Roberts, 1999). A switch from one D-type cyclin to another can be used to adjust the inputs that control proliferation, for example, when switching between cytokine-dependent and pre–T cell receptor–dependent proliferation in thymocytes (Sicinska et al., 2003). Although BCL6 had been identified as a plausible mediator of cyclin D2 downregulation in GC B cells (Shaffer et al., 2000), the signaling requirements for cyclin D3 upregulation have remained unclear. We now show that IL-21 represents a key trigger for cyclin D3 induction in GC B cells. While a recent study did not detect changes in cyclin D3 mRNA in antigen-specific GC B cells from IL-21R−/− mice (Zotos et al., 2021), this could potentially be because cyclin D3 is regulated post-translationally (Cato et al., 2011); indeed, cyclin D3 mRNA levels have been shown to be broadly similar between light zone and dark zone GC B cells despite substantially higher protein expression in the dark zone (Pae et al., 2021). Given that IL-21 can markedly upregulate global protein translation (Chou et al., 2016), it is tempting to speculate that this may underpin its ability to upregulate cyclin D3 protein without detectably changing mRNA levels.

Of note, the extent of dark zone loss in our IL-21R−/− mice shows striking similarity to that seen after complete loss of cyclin D3, where ∼70% of GC B cells retain a light zone phenotype (Pae et al., 2021). Similarly, in an independent study, the proportion of IL-21R−/− GC B cells expressing light zone markers in established GC was ∼70% (Zotos et al., 2021). We, therefore, postulate that IL-21 is required for most, if not all, of the cyclin D3 activity that drives inertial proliferation. This places IL-21 as a key component of the T cell–dependent light zone imprinting that tunes subsequent dark zone inertial proliferation. In contrast, IL-4 does not appear to play an equivalent role in directing GC dark zone proliferation. While elegant studies have shown that IL-21R−/− B cells exhibit reduced cell cycle entry, re-entry, and S-phase speed (Zotos et al., 2021; Dvorscek et al., 2022), in mice lacking both IL-4 and IL-13, GC B cell proliferation was actually elevated compared with wild-type controls, and the small GC present showed marked skewing to a dark zone phenotype (Turqueti-Neves et al., 2014). Consistent with this, treatment of mice with exogenous IL-4 (complexed with anti–IL-4 antibody) increased the frequency of GC light zone cells (Duan et al., 2021). Since the quality of T cell help controls the speed at which cells transit through cell cycle (Gitlin et al., 2015), as well as dark zone dwell time, the relative roles of IL-21 and IL-4 in dark zone homeostasis remain of interest, particularly as the representation of these cytokines changes over time during the GC response (Weinstein et al., 2016; Gonzalez et al., 2018).

Interestingly, emerging evidence suggests that STAT3−/− mice exhibit a similar pattern of light zone skewing to that seen in IL-21R deficiency (Fike et al., 2022 *Preprint*), consistent with the ability of IL-21 to activate STAT3 in mice and humans (Zeng et al., 2007; Good et al., 2006). However, the phenotypes of STAT3-deficient and IL-21R–deficient mice are distinct, with the former showing minimal differences in GC B cell proliferation and no significant difference in c-Myc expression at the population level compared with wild-type animals. Since IL-21 is not the only stimulus capable of activating STAT3, and can also modulate B cell responses via alternative pathways including PI3K (Attridge et al., 2014), the lack of complete overlap between phenotypes is not unexpected.

Foxo1 has been shown to promote transcription of an array of GC dark zone–associated target genes including cyclin D3 (Dominguez-Sola et al., 2015; Sander et al., 2015). We now show that IL-21 can modulate Foxo1 expression in GC B cells in vivo and that IL-21–dependent upregulation of cyclin D3 expression is at least in part dependent on the Foxo1 pathway, providing a novel link between IL-21 signaling and GC dark zone formation and maintenance. Work in CD4 T cells has implicated STAT-3–activating cytokines in promoting Foxo1 nuclear import (Oh et al., 2012) and our confocal microscopy analysis demonstrates the potential for IL-21 to aid Foxo1 nuclear translocation in IgM-stimulated B cells. Further studies will be required to elucidate the relative contribution of IL-21–mediated Foxo1 upregulation and nuclear translocation to the zonal organization of GC in vivo.

T cell help is unquestionably a key arbiter of B cell fate choice within the GC. However, recent work from the Bannard group has challenged the notion that T cells act as a limiting factor in controlling GC B cell cyclic re-entry. Rather than a set threshold of T cell help being required for re-entry of selected light zone GC B cells into the cell cycle, the authors instead propose that the amount of T cell help provided (reflecting B cell affinity) influences the division capacity in the dark zone (Long et al., 2022). Thus, T cells are not the gatekeepers of light zone GC B cell cyclic re-entry but rather determine the extent to which B cells are “refueled” for their subsequent expansion in the dark zone. Molecular players implicated in refueling include BATF and mTORC1, both established targets of IL-21 signaling (Inoue et al., 2017; Ersching et al., 2017; Xin et al., 2015; Kato and Perl, 2018). Together with its ability to promote c-Myc and cyclin D3 upregulation as shown here, as well as its capacity to sustain expression of AP4 (Chou et al., 2016), IL-21 emerges as a strong candidate to direct the metabolic reprogramming that fuels GC B cells for dark zone proliferation.

The roles of IL-21 identified in our study could potentially be relevant in autoimmune settings where this cytokine is frequently overproduced (Long et al., 2019; Spolski and Leonard, 2014). Increased frequencies of IL-21–producing Tfh cells (Kenefeck et al., 2015; Ferreira et al., 2015) could allow selection of B cells at a lower threshold of CD40 engagement and enhance cyclin D3 expression, potentially supporting the expansion, mutation, and differentiation of self-reactive B cell clones. Of note, the frequency of light zone GC B cells with a c-Myc+BATF+IRF4+ selected phenotype was markedly higher in autoimmune GC in CTLA-4–deficient mice compared with immunization-induced GC in CTLA-4–sufficient animals. Tfh are intrinsically “stingy” producers of IL-21 (Dan et al., 2016; Havenar-Daughton et al., 2016) and IL-21 levels drop after the first 2 d of the GC response (Zhang et al., 2018), suggesting competition for GC B cell refueling may increase with time. Overproduction of IL-21 may interfere with this, resulting in the participation and promotion of B cell clones that would otherwise be outcompeted. Indeed, a recent study from the D. Yu laboratory demonstrated that exogenous IL-21 markedly reduced GC B cell selection stringency (Chen et al., 2023). Intriguingly, recent findings suggest that IL-21 can also act in a non-cognate paracrine fashion (Quast et al., 2022), increasing the potential for excess IL-21 to influence humoral immunity in a bystander manner. Finally, since injection of anti–CTLA-4 antibodies can also promote IL-21 production (Wang et al., 2015), our findings may be relevant to autoimmune adverse events in cancer patients receiving checkpoint immunotherapy, where autoantibodies frequently emerge (Tahir et al., 2019; Ghosh et al., 2022).

## Materials and methods

### Mice

BALB/c mice were purchased from The Jackson Laboratory. Rag2−/− mice were purchased from Taconic Farms. CTLA-4−/− mice on a BALB/c background were kindly provided by A. Sharpe (Harvard, Boston, MA, USA). CTLA-4−/− mice were maintained on a rag2−/− background and crossed with CTLA-4+/−rag2+/− mice to generate CTLA-4−/−rag2+/− and CTLA-4+/−rag2+/− offspring. IL-21R−/− mice were kindly provided by Manfred Kopf (ETH Zurich, Zurich, Switzerland). IL-21R−/− mice were crossed with CTLA-4−/−rag2−/− mice to generate IL-21R−/−CTLA-4−/−rag2+ mice. Mice were housed in individually ventilated cages in a temperature- and humidity-controlled facility with a 14-h light and 10-h dark cycle and ad libitum feeding at a University College London Biological Services Unit. Mice were provided with environmental enrichment including cardboard tunnels, paper houses, chewing blocks, and aspen wood wool nesting material. Experiments were performed in accordance with the relevant Home Office project and personal licenses following approval from the University College London Animal Welfare Ethical Review Body.

### In vivo experiments

For the generation of T cell–dependent GC responses, 6–15-wk-old male and female mice were immunized intraperitoneally (i.p.) with 2 × 10^9^ SRBC (TCS Biosciences). When indicated, anti-CD40L blocking antibodies (clone MR-1; Bio X Cell) were injected i.p. 5.5 and 6.5 d after SRBC immunization (200 µg per injection), and agonistic anti-CD40 antibodies (clone FGK4.5; Bio X Cell) were injected 5 d after SRBC immunization (50 µg per injection). For the generation of bone marrow chimeric mice, 7–11-wk-old male and female rag2−/− recipients were sublethally irradiated with 2 Gray, intravenously injected with 5 × 10^6^ bone marrow cells from wild-type and IL-21R−/− donor mice prepared at a one-to-one ratio; control animals received 5 × 10^6^ bone marrow cells from either wild-type or IL-21R−/− donors. Bone marrow recipients were allowed to reconstitute for at least 8 wk before i.p. immunization with 2 × 10^9^ SRBC.

All injections were carried out in the absence of anesthesia and analgesia, and mice were returned to their home cages immediately following the procedures. Most injections were performed in the morning. The welfare of experimental animals was monitored regularly (typically immediately after procedure, then at least every 2–3 d). No adverse events were noted during these experiments.

### Short-term B cell cultures

Splenic CD19^+^ B cells from BALB/c mice were purified using magnetic mouse CD19 MicroBeads (Miltenyi Biotec) and cultured in flat-bottom 96-well plates (Corning). To assess effects of IL-21 on the expression of c-Myc, BATF, IRF4, p-S6, cyclin D3, and Foxo1, cells were incubated with the indicated concentration of IL-21 (PeproTech) and 20 μg/ml of anti-mouse CD40 (FGK4.5) for 24 h at 37°C. To inhibit Foxo1, cells were treated with AS1842856 (2 μM; MedChem Express) for 1 h, followed by further incubation with 20 μg/ml of anti-CD40 mAb with or without 80 ng/ml of IL-21 for 16 h. To study Foxo1 intracellular localization, cells were rested for 30 min at 37°C, and where indicated, further incubated with 10 μg/ml of goat anti-mouse IgM (μ chain specific; Thermo Fisher Scientific) for 10 min followed by a 30-min stimulation with 80 ng/ml of IL-21.

### Flow cytometry

Mouse spleens and thymuses were mashed to obtain a single-cell suspension, and splenic red blood cells were lysed using ammonium-chloride-potassium lysis buffer. 2 × 10^6^ cells per sample were stained in round-bottom flow cytometry tubes. To discriminate between live and dead cells, samples were stained with Fixable Viability Dye eFluor 780 (Thermo Fisher Scientific). To limit non-specific antibody binding to Fc receptors, samples were preincubated with purified anti-CD16 (FcγRIII)/CD32 (FcγRII) antibodies (2.4G2; BD Biosciences). Cells were stained with surface antibodies against CD3 (17A2), CD3 (145-2C11), CD4 (GK1.5), CD4 (RM4-5), CD8 (53-6.7), CD19 (1D3; BD Biosciences), CD25 (PC61.5), CD44 (IM7), CD86 (GL-1; BioLegend), CD138 (281-2; BioLegend), CXCR4 (CD184; 2B11), CXCR5 (CD185; L138D7; BioLegend), Fas (CD95; Jo2; BD Biosciences), GL-7 (GL7; BioLegend), IgD (1-26c [11-26]), IL-21R (CD360; eBio4A9), PD-1 (CD279; RMP1-30; BioLegend), and Thy1.1 (CD90.1; HIS51). Antibodies purchased from Thermo Fisher Scientific unless otherwise stated. For staining of intracellular antigens BATF (D7C5; Cell Signaling Technology), BCL6 (7D1; BioLegend), c-Myc (D84C12; Cell Signaling Technology and Y69; abcam), FoxP3 (FJK-16s; Thermo Fisher Scientific), IRF4 (3E4; Thermo Fisher Scientific), and p-S6 (Ser235/236; D57.2.2E; Cell Signaling Technology), cells were fixed and permeabilized using the Foxp3/Transcription Factor Staining Buffer Set (Thermo Fisher Scientific) following the manufacturer’s instructions. For staining of intracellular cyclin D3 (DCS-22; BioLegend) and Foxo1 (C29H4; Cell Signaling Technology), cells were fixed with 2% paraformaldehyde (PFA; Sigma-Aldrich) and permeabilized using 0.1% Triton X-100 buffer (Sigma-Aldrich).

### Flow cytometry data acquisition and analysis

Samples were acquired on a BD LSRFortessa flow cytometer using the FACSDiva acquisition software (BD Biosciences). Fluorochrome spillover compensation was performed using UltraComp eBeads (Thermo Fisher Scientific). Analyses were performed using FlowJo software version 10 (BD Biosciences). For t-distributed stochastic neighbor embedding (t-SNE) plots, FlowJo data were loaded into R using the Bioconductor package flowCore, and plots were generated using CRAN packages Rtsne and ggplot2.

### Confocal microscopy

Mouse spleens were frozen and 7-μm tissue sections were prepared using a Bright OTF5000 Cryostat. For stains including anti-BCL6 antibodies, sections were fixed with 4% PFA and permeabilized using a saponin wash buffer (PBS supplemented with 0.5% saponin [Sigma-Aldrich]). Sections were stained with mouse anti-BCL6 (K112-91; Alexa Fluor 488; BD Biosciences) followed by rabbit anti–Alexa Fluor 488 (polyclonal Igs; Thermo Fisher Scientific) and donkey anti-rabbit (polyclonal Igs; Alexa Fluor 488; Thermo Fisher Scientific) antibodies; hamster anti-CD3 (145-2C11; BD Biosciences) followed by goat anti-hamster (polyclonal Igs; DyLight 405 or Cy5; Jackson ImmunoResearch) antibodies; rat anti-IgD (11-26c (11-26); FITC or eFluor 450; Thermo Fisher Scientific) antibodies; and rat anti-CD35 (8C12; biotin; BD Biosciences) followed by mouse anti-biotin (3D6.6; TRITC; Jackson ImmunoResearch) antibodies. For stains including anti-active caspase 3 antibodies, sections were fixed with acetone and stained with rabbit anti-active caspase 3 (C92-605; BD Biosciences) followed by donkey anti-rabbit (polyclonal Igs; Alexa Fluor 647; Thermo Fisher Scientific) antibodies and anti-CD3, anti-CD35, and anti-IgD antibodies as described above. EdU incorporation was detected using the Click-iT EdU Alexa Fluor 647 Flow Cytometry Assay Kit (Thermo Fisher Scientific) following the manufacturer’s instructions. Coverslips were mounted using ProLong Gold Antifade Mountant (Thermo Fisher Scientific).

For single-cell analysis, cells were transferred to poly-L-lysine coated glass-bottom 96-well plates (Sensopate; Greiner Bio-One) and fixed with 4% PFA. Non-specific binding was blocked using 5% bovine serum albumin buffer and cells were stained with anti-IgD (11-26c (11-26); biotin) followed by streptavidin (Alexa Fluor 555; Thermo Fisher Scientific). For staining of Foxo1 (C29H4; Alexa Fluor 647; Cell Signaling Technology), cells were permeabilized using 0.1% Triton X-100 buffer. Samples were stained with 2 µg/ml of DAPI (Sigma-Aldrich) and mounted in Mowiol with 2.5% 1,4-diazabicyclo(2.2.2)octane.

### Image acquisition and analysis

Images were acquired on a Nikon C2 confocal microscope using NIS-Elements software (Nikon). Image processing and analysis were performed using FIJI (ImageJ) and CellProfiler version 2.2.0 software packages.

### Western blotting

Cells were seeded in 12-well plates at 2 × 10^6^ cells/ml and treated with 80 ng/ml of IL-21 and/or 20 μg/ml of anti-mouse CD40 (FGK4.5) where indicated. After 24 h at 37°C, cells were washed with cold PBS and lysed using a modified radioimmunoprecipitation assay lysis buffer (50 mM Tris-HCl, pH 7.4, 150 mM NaCl, 2 mM EDTA, 2 mM Na-pyrophosphate, 50 mM NaF, 1% Triton X-100, 0.5% sodium deoxycholate, 1% sodium orthovanadate, and 0.1% SDS) containing cOmplete ULTRA protease inhibitor cocktail (Roche), left on ice for 30 min, and then centrifuged for 10 min at 13,000 revolutions per minute at 4°C. Cleared total protein lysate content was determined using the BCA protein assay kit (Thermo Fisher Scientific). Subsequently, 2 × Laemmli sample buffer (Bio-Rad) was added to 25 μg of total protein with a final dilution of 1:40 β-mercaptoethanol, followed by boiling at 95°C for 5 min. Resulting samples were added to 4–20% Tris-Glycine gels (Bio-Rad) and separated by SDS-PAGE before transfer to fluorescent polyvinylidene difluoride membranes (Merck Millipore) and blocking for 1 h with LI-COR blocking buffer. Membranes were incubated with mouse anti-cyclin D3 primary antibody (DCS22; Cell Signalling Technology) in blocking buffer with 0.2% Tween 20 overnight at 4°C. Subsequently, membranes were washed with TBS-T and probed with IRDye 680RD goat anti-mouse IgG secondary antibodies (LI-COR) in a blocking buffer with 0.2% Tween 20 for 1 h before being washed again and imaged using the LI-COR Bioscience Odyssey M Imager. α Tubulin was used as a loading control; tubulin was detected using mouse anti–α tubulin primary antibody (Sigma-Aldrich) and probed simultaneously with cyclin D3 using the same secondary antibody. Quantification of band density was conducted using the Empiria Studio software.

### CaspGLOW apoptosis assay

Apoptosis was measured using the Thermo Fisher Scientific CaspGLOW Fluorescein Active Caspase Staining Kit. Immediately following cell isolation, splenocytes were incubated with Z-VAD-FMK-FITC for 45 min at 37°C. Cells were washed according to the manufacturer’s instructions and stained with a fixable viability dye and antibodies against surface antigens as described above.

### Assessment of in vivo cell proliferation

For in vivo cell proliferation analysis, mice were injected i.p. with 2 mg of EdU (Thermo Fisher Scientific) 30 min before tissue harvest. Cells from mice treated with EdU were processed and stained with antibodies against surface antigens as described above. EdU incorporation was detected using the Click-iT EdU Alexa Fluor 647 Flow Cytometry Assay Kit (Thermo Fisher Scientific) following the manufacturer’s instructions. Before sample acquisition, cells were stained with 7-amino-actinomycin D.

### Statistical analyses

Numerical data were visualized, and statistical analyses were performed using GraphPad Prism (GraphPad Software Inc.). Analysis of independent sample sets was performed using two-tailed Mann–Whitney U tests. Analysis of paired samples was performed using two-tailed Wilcoxon matched-pairs signed-rank tests. Comparisons of more than two groups of samples were performed using two-sided Kruskal–Wallis tests or one-way ANOVA. Statistical significance was denoted as ****, P < 0.0001; ***, P < 0.001; **, P < 0.01; *, P < 0.05; ns, not significant.

### Online supplemental material

Fig. S1 shows the effects of IL-21 on GC size and polarization in CTLA-4−/− and immunized wild-type mice. Fig. S2 demonstrates comparable caspase activity in GC from immunized wild-type and IL-21R−/− mice. Fig. S3 shows that c-Myc+BATF+IRF4+ light zone GC B cell formation in vivo can be regulated by CD40 signaling. This figure also demonstrates that this light zone GC B cell subset expresses high levels of p-S6 and that IL-21 can modulate p-S6 expression in B cells. Lastly, this figure demonstrates that IL-21 promotes c-Myc+BATF+IRF4+ light zone GC B cell formation in CTLA-4−/− mice. Fig. S4 shows validation of flow cytometry staining with cyclin D3 antibody clone DCS-22 using wild-type thymocytes. Fig. S5 demonstrates that IL-21 promotes proliferation of GC B cells in immunized wild-type mice.

## Supplementary Material

SourceData F5 is the source file for Fig. 5.

## Data Availability

Data are available in the article or upon a reasonable request to the corresponding author.

## References

[bib1] Allen, C.D.C.C., K.M. Ansel, C. Low, R. Lesley, H. Tamamura, N. Fujii, and J.G. Cyster. 2004. Germinal center dark and light zone organization is mediated by CXCR4 and CXCR5. Nat. Immunol. 5:943–952. 10.1038/ni1100 15300245

[bib2] Asao, H., C. Okuyama, S. Kumaki, N. Ishii, S. Tsuchiya, D. Foster, and K. Sugamura. 2001. Cutting edge: The common γ-chain is an indispensable subunit of the IL-21 receptor complex. J. Immunol. 167:1–5. 10.4049/jimmunol.167.1.1 11418623

[bib3] Attridge, K., R. Kenefeck, L. Wardzinski, O.S. Qureshi, C.J. Wang, C. Manzotti, K. Okkenhaug, and L.S.K. Walker. 2014. IL-21 promotes CD4 T cell responses by phosphatidylinositol 3-kinase-dependent upregulation of CD86 on B cells. J. Immunol. 192:2195–2201. 10.4049/jimmunol.1302082 24470500 PMC3932810

[bib4] Bannard, O., R.M. Horton, C.D.C.C. Allen, J. An, T. Nagasawa, and J.G. Cyster. 2013. Germinal center centroblasts transition to a centrocyte phenotype according to a timed program and depend on the dark zone for effective selection. Immunity. 39:912–924. 10.1016/j.immuni.2013.08.038 24184055 PMC3828484

[bib5] Bessa, J., M. Kopf, and M.F. Bachmann. 2010. Cutting edge: IL-21 and TLR signaling regulate germinal center responses in a B cell-intrinsic manner. J. Immunol. 184:4615–4619. 10.4049/jimmunol.0903949 20368279

[bib6] Calado, D.P., Y. Sasaki, S.A. Godinho, A. Pellerin, K. Köchert, B.P. Sleckman, I.M. de Alborán, M. Janz, S. Rodig, and K. Rajewsky. 2012. The cell-cycle regulator c-Myc is essential for the formation and maintenance of germinal centers. Nat. Immunol. 13:1092–1100. 10.1038/ni.2418 23001146 PMC4132664

[bib7] Cato, M.H., S.K. Chintalapati, I.W. Yau, S.A. Omori, and R.C. Rickert. 2011. Cyclin D3 is selectively required for proliferative expansion of germinal center B cells. Mol. Cell. Biol. 31:127–137. 10.1128/mcb.00650-10 20956554 PMC3019862

[bib8] Chen, Z., Y. Cui, Y. Yao, B. Liu, J. Yunis, X. Gao, N. Wang, P.F. Cañete, Z.K. Tuong, H. Sun, . 2023. Heparan sulfate regulates IL-21 bioavailability and signal strength that control germinal center B cell selection and differentiation. Sci. Immunol. 8:eadd1728. 10.1126/sciimmunol.add1728 36800411

[bib9] Chou, C., D.J. Verbaro, E. Tonc, M. Holmgren, M. Cella, M. Colonna, D. Bhattacharya, and T. Egawa. 2016. The transcription factor AP4 mediates resolution of chronic viral infection through amplification of germinal center B cell responses. Immunity. 45:570–582. 10.1016/j.immuni.2016.07.023 27566940 PMC5037962

[bib10] Chtanova, T., S.G. Tangye, R. Newton, N. Frank, M.R. Hodge, M.S. Rolph, and C.R. Mackay. 2004. T follicular helper cells express a distinctive transcriptional profile, reflecting their role as non-Th1/Th2 effector cells that provide help for B cells. J. Immunol. 173:68–78. 10.4049/jimmunol.173.1.68 15210760

[bib11] Collins, C.M., and S.H. Speck. 2015. Interleukin 21 signaling in B cells is required for efficient establishment of murine gammaherpesvirus latency. PLoS Pathog. 11:e1004831. 10.1371/journal.ppat.1004831 25875847 PMC4395336

[bib12] Dan, J.M., C.S. Lindestam Arlehamn, D. Weiskopf, R. da Silva Antunes, C. Havenar-Daughton, S.M. Reiss, M. Brigger, M. Bothwell, A. Sette, and S. Crotty. 2016. A cytokine-independent approach to identify antigen-specific human germinal center T follicular helper cells and rare antigen-specific CD4^+^ T cells in blood. J. Immunol. 197:983–993. 10.4049/jimmunol.1600318 27342848 PMC4955771

[bib13] Dolff, S., W.H. Abdulahad, J. Westra, B. Doornbos-van der Meer, P.C. Limburg, C.G.M. Kallenberg, and M. Bijl. 2011. Increase in IL-21 producing T-cells in patients with systemic lupus erythematosus. Arthritis Res. Ther. 13:R157. 10.1186/ar3474 21959034 PMC3308088

[bib14] Dominguez-Sola, D., J. Kung, A.B. Holmes, V.A. Wells, T. Mo, K. Basso, and R. Dalla-Favera. 2015. The FOXO1 transcription factor instructs the germinal center dark zone program. Immunity. 43:1064–1074. 10.1016/j.immuni.2015.10.015 26620759

[bib15] Dominguez-Sola, D., G.D. Victora, C.Y. Ying, R.T. Phan, M. Saito, M.C. Nussenzweig, and R. Dalla-Favera. 2012. The proto-oncogene MYC is required for selection in the germinal center and cyclic reentry. Nat. Immunol. 13:1083–1091. 10.1038/ni.2428 23001145 PMC3711534

[bib16] Duan, L., D. Liu, H. Chen, M.A. Mintz, M.Y. Chou, D.I. Kotov, Y. Xu, J. An, B.J. Laidlaw, and J.G. Cyster. 2021. Follicular dendritic cells restrict interleukin-4 availability in germinal centers and foster memory B cell generation. Immunity. 54:2256–2272.e6. 10.1016/j.immuni.2021.08.028 34555336 PMC8516727

[bib17] Dvorscek, A.R., C.I. McKenzie, M.J. Robinson, Z. Ding, C. Pitt, K. O’Donnell, D. Zotos, R. Brink, D.M. Tarlinton, and I. Quast. 2022. IL-21 has a critical role in establishing germinal centers by amplifying early B cell proliferation. EMBO Rep. 23:e54677. 10.15252/embr.202254677 35801309 PMC9442303

[bib18] Elsaesser, H., K. Sauer, and D.G. Brooks. 2009. IL-21 is required to control chronic viral infection. Science. 324:1569–1572. 10.1126/science.1174182 19423777 PMC2830017

[bib19] Erman, B., I. Bilic, T. Hirschmugl, E. Salzer, D. Çagdas, S. Esenboga, Z. Akcoren, O. Sanal, I. Tezcan, and K. Boztug. 2015. Combined immunodeficiency with CD4 lymphopenia and sclerosing cholangitis caused by a novel loss-of-function mutation affecting IL21R. Haematologica. 100:e216–e219. 10.3324/haematol.2014.120980 25769540 PMC4450632

[bib20] Ersching, J., A. Efeyan, L. Mesin, J.T. Jacobsen, G. Pasqual, B.C. Grabiner, D. Dominguez-Sola, D.M. Sabatini, and G.D. Victora. 2017. Germinal center selection and affinity maturation require dynamic regulation of mTORC1 kinase. Immunity. 46:1045–1058.e6. 10.1016/j.immuni.2017.06.005 28636954 PMC5526448

[bib21] Eto, D., C. Lao, D. DiToro, B. Barnett, T.C. Escobar, R. Kageyama, I. Yusuf, and S. Crotty. 2011. IL-21 and IL-6 are critical for different aspects of B cell immunity and redundantly induce optimal follicular helper CD4 T cell (Tfh) differentiation. PLoS One. 6:e17739. 10.1371/journal.pone.0017739 21423809 PMC3056724

[bib22] Ferguson, S.E., S. Han, G. Kelsoe, and C.B. Thompson. 1996. CD28 is required for germinal center formation. J. Immunol. 156:4576–4581. 8648099

[bib23] Ferreira, R.C., H.Z. Simons, W.S. Thompson, A.J. Cutler, X.C. Dopico, D.J. Smyth, M. Mashar, H. Schuilenburg, N.M. Walker, D.B. Dunger, . 2015. IL-21 production by CD4^+^ effector T cells and frequency of circulating follicular helper T cells are increased in type 1 diabetes patients. Diabetologia. 58:781–790. 10.1007/s00125-015-3509-8 25652388 PMC4351433

[bib24] Fike, A.J., S.B. Chodisetti, N.E. Wright, K.N. Bricker, P.P. Domeier, M. Maienschein-Cline, A.M. Rosenfeld, S.A. Luckenbill, J.L. Weber, N.M. Choi, . 2022. STAT3 signaling in B cells controls germinal center zone organization and recycling. bioRxiv. 10.1101/2022.08.12.503811 (Preprint posted August 12, 2022). PMC10311431 37200190

[bib25] Finkin, S., H. Hartweger, T.Y. Oliveira, E.E. Kara, and M.C. Nussenzweig. 2019. Protein amounts of the MYC transcription factor determine germinal center B cell division capacity. Immunity. 51:324–336.e5. 10.1016/j.immuni.2019.06.013 31350178 PMC6703930

[bib26] Ghosh, N., K.K. Chan, B. Jivanelli, and A.R. Bass. 2022. Autoantibodies in patients with immune-related adverse events from checkpoint inhibitors: A systematic literature review. J. Clin. Rheumatol. 28:e498–e505. 10.1097/RHU.0000000000001777 34371516 PMC8816970

[bib28] Gitlin, A.D., Z. Shulman, and M.C. Nussenzweig. 2014. Clonal selection in the germinal centre by regulated proliferation and hypermutation. Nature. 509:637–640. 10.1038/nature13300 24805232 PMC4271732

[bib27] Gitlin, A.D., C.T. Mayer, T.Y. Oliveira, Z. Shulman, M.J.K. Jones, A. Koren, and M.C. Nussenzweig. 2015. T cell help controls the speed of the cell cycle in germinal center B cells. Science. 349:643–646. 10.1126/science.aac4919 26184917 PMC4809261

[bib29] Gonzalez, D.G., C.M. Cote, J.R. Patel, C.B. Smith, Y. Zhang, K.M. Nickerson, T. Zhang, S.M. Kerfoot, and A.M. Haberman. 2018. Nonredundant roles of IL-21 and IL-4 in the phased initiation of germinal center B cells and subsequent self-renewal transitions. J. Immunol. 201:3569–3579. 10.4049/jimmunol.1500497 30446568 PMC6289626

[bib30] Good, K.L., V.L. Bryant, and S.G. Tangye. 2006. Kinetics of human B cell behavior and amplification of proliferative responses following stimulation with IL-21. J. Immunol. 177:5236–5247. 10.4049/jimmunol.177.8.5236 17015709

[bib31] Good-Jacobson, K.L., E. Song, S. Anderson, A.H. Sharpe, and M.J. Shlomchik. 2012. CD80 expression on B cells regulates murine T follicular helper development, germinal center B cell survival, and plasma cell generation. J. Immunol. 188:4217–4225. 10.4049/jimmunol.1102885 22450810 PMC3331930

[bib32] Good-Jacobson, K.L., C.G. Szumilas, L. Chen, A.H. Sharpe, M.M. Tomayko, and M.J. Shlomchik. 2010. PD-1 regulates germinal center B cell survival and the formation and affinity of long-lived plasma cells. Nat. Immunol. 11:535–542. 10.1038/ni.1877 20453843 PMC2874069

[bib33] Gurwicz, N., L. Stoler-Barak, N. Schwan, A. Bandyopadhyay, M. Meyer-Hermann, and Z. Shulman. 2023. Tingible body macrophages arise from lymph node-resident precursors and uptake B cells by dendrites. J. Exp. Med. 220:e20222173. 10.1084/jem.20222173 36705667 PMC9900388

[bib34] Habib, T., S. Senadheera, K. Weinberg, and K. Kaushansky. 2002. The common γ chain (γ c) is a required signaling component of the IL-21 receptor and supports IL-21-induced cell proliferation via JAK3. Biochemistry. 41:8725–8731. 10.1021/bi0202023 12093291

[bib35] Havenar-Daughton, C., S.M. Reiss, D.G. Carnathan, J.E. Wu, K. Kendric, A. Torrents de la Peña, S.P. Kasturi, J.M. Dan, M. Bothwell, R.W. Sanders, . 2016. Cytokine-independent detection of antigen-specific germinal center T follicular helper cells in immunized nonhuman primates using a live cell activation-induced marker technique. J. Immunol. 197:994–1002. 10.4049/jimmunol.1600320 27335502 PMC4955744

[bib36] Inoue, T., R. Shinnakasu, W. Ise, C. Kawai, T. Egawa, and T. Kurosaki. 2017. The transcription factor Foxo1 controls germinal center B cell proliferation in response to T cell help. J. Exp. Med. 214:1181–1198. 10.1084/jem.20161263 28351982 PMC5379976

[bib37] Karnowski, A., S. Chevrier, G.T. Belz, A. Mount, D. Emslie, K. D’Costa, D.M. Tarlinton, A. Kallies, and L.M. Corcoran. 2012. B and T cells collaborate in antiviral responses via IL-6, IL-21, and transcriptional activator and coactivator, Oct2 and OBF-1. J. Exp. Med. 209:2049–2064. 10.1084/jem.20111504 23045607 PMC3478936

[bib38] Kato, H., and A. Perl. 2018. Blockade of treg cell differentiation and function by the interleukin-21-mechanistic target of rapamycin axis via suppression of autophagy in patients with systemic lupus erythematosus. Arthritis Rheumatol. 70:427–438. 10.1002/art.40380 29161463 PMC5826851

[bib39] Kenefeck, R., C.J. Wang, T. Kapadi, L. Wardzinski, K. Attridge, L.E. Clough, F. Heuts, A. Kogimtzis, S. Patel, M. Rosenthal, . 2015. Follicular helper T cell signature in type 1 diabetes. J. Clin. Invest. 125:292–303. 10.1172/JCI76238 25485678 PMC4382272

[bib40] Ketzer, F., H. Abdelrasoul, M. Vogel, R. Marienfeld, M. Müschen, H. Jumaa, T. Wirth, and A. Ushmorov. 2022. CCND3 is indispensable for the maintenance of B-cell acute lymphoblastic leukemia. Oncogenesis. 11:1. 10.1038/s41389-021-00377-0 35013097 PMC8748974

[bib41] Kim, H.P., L.L. Korn, A.M. Gamero, and W.J. Leonard. 2005. Calcium-dependent activation of interleukin-21 gene expression in T cells. J. Biol. Chem. 280:25291–25297. 10.1074/jbc.M501459200 15879595

[bib42] Kotlarz, D., N. Ziętara, G. Uzel, T. Weidemann, C.J. Braun, J. Diestelhorst, P.M. Krawitz, P.N. Robinson, J. Hecht, J. Puchałka, . 2013. Loss-of-function mutations in the IL-21 receptor gene cause a primary immunodeficiency syndrome. J. Exp. Med. 210:433–443. 10.1084/jem.20111229 23440042 PMC3600901

[bib43] Laidlaw, B.J., L. Duan, Y. Xu, S.E. Vazquez, and J.G. Cyster. 2020. The transcription factor Hhex cooperates with the corepressor Tle3 to promote memory B cell development. Nat. Immunol. 21:1082–1093. 10.1038/s41590-020-0713-6 32601467 PMC7442689

[bib44] Linterman, M.A., L. Beaton, D. Yu, R.R. Ramiscal, M. Srivastava, J.J. Hogan, N.K. Verma, M.J. Smyth, R.J. Rigby, and C.G. Vinuesa. 2010. IL-21 acts directly on B cells to regulate Bcl-6 expression and germinal center responses. J. Exp. Med. 207:353–363. 10.1084/jem.20091738 20142429 PMC2822609

[bib45] Liu, R., Q. Wu, D. Su, N. Che, H. Chen, L. Geng, J. Chen, W. Chen, X. Li, and L. Sun. 2012. A regulatory effect of IL-21 on T follicular helper-like cell and B cell in rheumatoid arthritis. Arthritis Res. Ther. 14:R255. 10.1186/ar4100 23176102 PMC3674600

[bib46] Long, D., Y. Chen, H. Wu, M. Zhao, and Q. Lu. 2019. Clinical significance and immunobiology of IL-21 in autoimmunity. J. Autoimmun. 99:1–14. 10.1016/j.jaut.2019.01.013 30773373

[bib47] Long, Z., B. Phillips, D. Radtke, M. Meyer-Hermann, and O. Bannard. 2022. Competition for refueling rather than cyclic reentry initiation evident in germinal centers. Sci. Immunol. 7:eabm0775. 10.1126/sciimmunol.abm0775 35275753 PMC7614495

[bib48] Luo, W., L. Conter, R.A. Elsner, S. Smita, F. Weisel, D. Callahan, S. Wu, M. Chikina, and M. Shlomchik. 2023. IL-21R signal reprogramming cooperates with CD40 and BCR signals to select and differentiate germinal center B cells. Sci. Immunol. 8:eadd1823. 10.1126/sciimmunol.add1823 36800413 PMC10206726

[bib49] Luo, W., F. Weisel, and M.J. Shlomchik. 2018. B cell receptor and CD40 signaling are rewired for synergistic induction of the c-myc transcription factor in germinal center B cells. Immunity. 48:313–326.e5. 10.1016/j.immuni.2018.01.008 29396161 PMC5821563

[bib50] Lüthje, K., A. Kallies, Y. Shimohakamada, G.T. Belz, A. Light, D.M. Tarlinton, and S.L. Nutt. 2012. The development and fate of follicular helper T cells defined by an IL-21 reporter mouse. Nat. Immunol. 13:491–498. 10.1038/ni.2261 22466669

[bib51] Mayer, C.T., A. Gazumyan, E.E. Kara, A.D. Gitlin, J. Golijanin, C. Viant, J. Pai, T.Y. Oliveira, Q. Wang, A. Escolano, . 2017. The microanatomic segregation of selection by apoptosis in the germinal center. Science. 358:eaao2602. 10.1126/science.aao2602 28935768 PMC5957278

[bib52] Mehta, D.S., A.L. Wurster, and M.J. Grusby. 2004. Biology of IL-21 and the IL-21 receptor. Immunol. Rev. 202:84–95. 10.1111/j.0105-2896.2004.00201.x 15546387

[bib53] Niu, X., D. He, X. Zhang, T. Yue, N. Li, J.Z. Zhang, C. Dong, and G. Chen. 2010. IL-21 regulates Th17 cells in rheumatoid arthritis. Hum. Immunol. 71:334–341. 10.1016/j.humimm.2010.01.010 20079789

[bib54] Oh, H.M., C.R. Yu, I. Dambuza, B. Marrero, and C.E. Egwuagu. 2012. STAT3 protein interacts with Class O Forkhead transcription factors in the cytoplasm and regulates nuclear/cytoplasmic localization of FoxO1 and FoxO3a proteins in CD4(+) T cells. J. Biol. Chem. 287:30436–30443. 10.1074/jbc.M112.359661 22761423 PMC3436293

[bib55] Ozaki, K., R. Spolski, R. Ettinger, H.P. Kim, G. Wang, C.F. Qi, P. Hwu, D.J. Shaffer, S. Akilesh, D.C. Roopenian, . 2004. Regulation of B cell differentiation and plasma cell generation by IL-21, a novel inducer of Blimp-1 and Bcl-6. J. Immunol. 173:5361–5371. 10.4049/jimmunol.173.9.5361 15494482

[bib56] Ozaki, K., R. Spolski, C.G. Feng, C.-F.F. Qi, J. Cheng, A. Sher, H.C. Morse III, C. Liu, P.L. Schwartzberg, and W.J. Leonard. 2002. A critical role for IL-21 in regulating immunoglobulin production. Science. 298:1630–1634. 10.1126/science.1077002 12446913

[bib57] Pae, J., J. Ersching, T.B.R. Castro, M. Schips, L. Mesin, S.J. Allon, J. Ordovas-Montanes, C. Mlynarczyk, A. Melnick, A. Efeyan, . 2021. Cyclin D3 drives inertial cell cycling in dark zone germinal center B cells. J. Exp. Med. 218:e20201699. 10.1084/jem.20201699 33332554 PMC7754672

[bib58] Peled, J.U., J.J. Yu, J. Venkatesh, E. Bi, B.B. Ding, M. Krupski-Downs, R. Shaknovich, P. Sicinski, B. Diamond, M.D. Scharff, and B.H. Ye. 2010. Requirement for cyclin D3 in germinal center formation and function. Cell Res. 20:631–646. 10.1038/cr.2010.55 20404856 PMC2997820

[bib59] Di Pietro, A., J. Polmear, L. Cooper, T. Damelang, T. Hussain, L. Hailes, K. O’Donnell, V. Udupa, T. Mi, S. Preston, . 2022. Targeting BMI-1 in B cells restores effective humoral immune responses and controls chronic viral infection. Nat. Immunol. 23:86–98. 10.1038/s41590-021-01077-y 34845392

[bib60] Quast, I., A.R. Dvorscek, C. Pattaroni, T.M. Steiner, C.I. McKenzie, C. Pitt, K. O’Donnell, Z. Ding, D.L. Hill, R. Brink, . 2022. Interleukin-21, acting beyond the immunological synapse, independently controls T follicular helper and germinal center B cells. Immunity. 55:1414–1430.e5. 10.1016/j.immuni.2022.06.020 35896116

[bib61] Ramezani-Rad, P., C. Chen, Z. Zhu, and R.C. Rickert. 2020. Cyclin D3 governs clonal expansion of dark zone germinal center B cells. Cell Rep. 33:108403. 10.1016/j.celrep.2020.108403 33207194 PMC7714654

[bib62] Rasheed, M.A.U., D.R. Latner, R.D. Aubert, T. Gourley, R. Spolski, C.W. Davis, W.A. Langley, S.-J. Ha, L. Ye, S. Sarkar, . 2013. Interleukin-21 is a critical cytokine for the generation of virus-specific long-lived plasma cells. J. Virol. 87:7737–7746. 10.1128/jvi.00063-13 23637417 PMC3700268

[bib63] Salek-Ardakani, S., Y.S. Choi, M. Rafii-El-Idrissi Benhnia, R. Flynn, R. Arens, S. Shoenberger, S. Crotty, M. Croft, and S. Salek-Ardakani. 2011. B cell-specific expression of B7-2 is required for follicular Th cell function in response to vaccinia virus. J. Immunol. 186:5294–5303. 10.4049/jimmunol.1100406 21441451 PMC3089765

[bib64] Salzer, E., A. Kansu, H. Sic, P. Májek, A. Ikincioğullari, F.E. Dogu, N.K. Prengemann, E. Santos-Valente, W.F. Pickl, I. Bilic, . 2014. Early-onset inflammatory bowel disease and common variable immunodeficiency-like disease caused by IL-21 deficiency. J. Allergy Clin. Immunol. 133:1651–1659.e12. 10.1016/j.jaci.2014.02.034 24746753

[bib65] Sander, S., V.T. Chu, T. Yasuda, A. Franklin, R. Graf, D.P. Calado, S. Li, K. Imami, M. Selbach, M. Di Virgilio, . 2015. PI3 kinase and FOXO1 transcription factor activity differentially control B cells in the germinal center light and dark zones. Immunity. 43:1075–1086. 10.1016/j.immuni.2015.10.021 26620760

[bib66] Shaffer, A.L., X. Yu, Y. He, J. Boldrick, E.P. Chan, and L.M. Staudt. 2000. BCL-6 represses genes that function in lymphocyte differentiation, inflammation, and cell cycle control. Immunity. 13:199–212. 10.1016/S1074-7613(00)00020-0 10981963

[bib67] Sherr, C.J., and J.M. Roberts. 1999. CDK inhibitors: Positive and negative regulators of G1-phase progression. Genes Dev. 13:1501–1512. 10.1101/gad.13.12.1501 10385618

[bib68] Shulman, Z., A.D. Gitlin, J.S. Weinstein, B. Lainez, E. Esplugues, R.A. Flavell, J.E. Craft, and M.C. Nussenzweig. 2014. Dynamic signaling by T follicular helper cells during germinal center B cell selection. Science. 345:1058–1062. 10.1126/science.1257861 25170154 PMC4519234

[bib69] Sicinska, E., I. Aifantis, L. Le Cam, W. Swat, C. Borowski, Q. Yu, A.A. Ferrando, S.D. Levin, Y. Geng, H. von Boehmer, and P. Sicinski. 2003. Requirement for cyclin D3 in lymphocyte development and T cell leukemias. Cancer Cell. 4:451–461. 10.1016/S1535-6108(03)00301-5 14706337

[bib70] Spolski, R., and W.J. Leonard. 2014. Interleukin-21: A double-edged sword with therapeutic potential. Nat. Rev. Drug Discov. 13:379–395. 10.1038/nrd4296 24751819

[bib71] Stepensky, P., B. Keller, O. Abuzaitoun, A. Shaag, B. Yaacov, S. Unger, M. Seidl, M. Rizzi, M. Weintraub, O. Elpeleg, and K. Warnatz. 2015. Extending the clinical and immunological phenotype of human interleukin-21 receptor deficiency. Haematologica. 100:e72–e76. 10.3324/haematol.2014.112508 25398835 PMC4803132

[bib72] Strengell, M., S. Matikainen, J. Sirén, A. Lehtonen, D. Foster, I. Julkunen, and T. Sareneva. 2003. IL-21 in synergy with IL-15 or IL-18 enhances IFN-γ production in human NK and T cells. J. Immunol. 170:5464–5469. 10.4049/jimmunol.170.11.5464 12759422

[bib73] Tahir, S.A., J. Gao, Y. Miura, J. Blando, R.S.S. Tidwell, H. Zhao, S.K. Subudhi, H. Tawbi, E. Keung, J. Wargo, . 2019. Autoimmune antibodies correlate with immune checkpoint therapy-induced toxicities. Proc. Natl. Acad. Sci. USA. 116:22246–22251. 10.1073/pnas.1908079116 31611368 PMC6825284

[bib74] Tangye, S.G., and C.S. Ma. 2020. Regulation of the germinal center and humoral immunity by interleukin-21. J. Exp. Med. 217:e20191638. 10.1084/jem.20191638 31821441 PMC7037251

[bib75] Terrier, B., N. Costedoat-Chalumeau, M. Garrido, G. Geri, M. Rosenzwajg, L. Musset, D. Klatzmann, D. Saadoun, and P. Cacoub. 2012. Interleukin 21 correlates with T cell and B cell subset alterations in systemic lupus erythematosus. J. Rheumatol. 39:1819–1828. 10.3899/jrheum.120468 22859347

[bib76] Turqueti-Neves, A., M. Otte, O. Prazeres da Costa, U.E. Höpken, M. Lipp, T. Buch, and D. Voehringer. 2014. B-cell-intrinsic STAT6 signaling controls germinal center formation. Eur. J. Immunol. 44:2130–2138. 10.1002/eji.201344203 24777733

[bib77] Victora, G.D., D. Dominguez-Sola, A.B. Holmes, S. Deroubaix, R. Dalla-Favera, and M.C. Nussenzweig. 2012. Identification of human germinal center light and dark zone cells and their relationship to human B-cell lymphomas. Blood. 120:2240–2248. 10.1182/blood-2012-03-415380 22740445 PMC3447782

[bib78] Victora, G.D., and M.C. Nussenzweig. 2022. Germinal centers. Annu. Rev. Immunol. 40:413–442. 10.1146/annurev-immunol-120419-022408 35113731

[bib79] Victora, G.D., T.A. Schwickert, D.R. Fooksman, A.O. Kamphorst, M. Meyer-Hermann, M.L. Dustin, and M.C. Nussenzweig. 2010. Germinal center dynamics revealed by multiphoton microscopy with a photoactivatable fluorescent reporter. Cell. 143:592–605. 10.1016/j.cell.2010.10.032 21074050 PMC3035939

[bib80] Vinuesa, C.G., M.C. Cook, C. Angelucci, V. Athanasopoulos, L. Rui, K.M. Hill, D. Yu, H. Domaschenz, B. Whittle, T. Lambe, . 2005. A RING-type ubiquitin ligase family member required to repress follicular helper T cells and autoimmunity. Nature. 435:452–458. 10.1038/nature03555 15917799

[bib81] Vinuesa, C.G., I. Sanz, M.C. Cook, I. Sanz, and M.C. Cook. 2009. Dysregulation of germinal centres in autoimmune disease. Nat. Rev. Immunol. 9:845–857. 10.1038/nri2637 19935804

[bib82] Walker, L.S.K., A. Gulbranson-Judge, S. Flynn, T. Brocker, C. Raykundalia, M. Goodall, R. Förster, M. Lipp, and P. Lane. 1999. Compromised OX40 function in CD28-deficient mice is linked with failure to develop CXC chemokine receptor 5-positive CD4 cells and germinal centers. J. Exp. Med. 190:1115–1122. 10.1084/jem.190.8.1115 10523609 PMC2195670

[bib83] Wang, C.J., F. Heuts, V. Ovcinnikovs, L. Wardzinski, C. Bowers, E.M. Schmidt, A. Kogimtzis, R. Kenefeck, D.M. Sansom, and L.S.K.K. Walker. 2015. CTLA-4 controls follicular helper T-cell differentiation by regulating the strength of CD28 engagement. Proc. Natl. Acad. Sci. USA. 112:524–529. 10.1073/pnas.1414576112 25548162 PMC4299196

[bib84] Wang, F., S. Demir, F. Gehringer, C.D. Osswald, F. Seyfried, S. Enzenmüller, S.M. Eckhoff, T. Maier, K. Holzmann, K.M. Debatin, . 2018. Tight regulation of FOXO1 is essential for maintenance of B-cell precursor acute lymphoblastic leukemia. Blood. 131:2929–2942. 10.1182/blood-2017-10-813576 29622548

[bib85] Weinstein, J.S., E.I. Herman, B. Lainez, P. Licona-Limón, E. Esplugues, R. Flavell, and J. Craft. 2016. TFH cells progressively differentiate to regulate the germinal center response. Nat. Immunol. 17:1197–1205. 10.1038/ni.3554 27573866 PMC5030190

[bib86] Xin, G., D.M. Schauder, B. Lainez, J.S. Weinstein, Z. Dai, Y. Chen, E. Esplugues, R. Wen, D. Wang, I.A. Parish, . 2015. A critical role of IL-21-induced BATF in sustaining CD8-T-cell-mediated chronic viral control. Cell Rep. 13:1118–1124. 10.1016/j.celrep.2015.09.069 26527008 PMC4859432

[bib87] Yam-Puc, J.C., L. Zhang, R.A. Maqueda-Alfaro, L. Garcia-Ibanez, Y. Zhang, J. Davies, Y.A. Senis, M. Snaith, and K.M. Toellner. 2021. Enhanced BCR signaling inflicts early plasmablast and germinal center B cell death. iScience. 24:102038. 10.1016/j.isci.2021.102038 33532715 PMC7822941

[bib88] Yoshida, N., D. Kitayama, M. Arima, A. Sakamoto, A. Inamine, H. Watanabe-Takano, M. Hatano, T. Koike, and T. Tokuhisa. 2011. CXCR4 expression on activated B cells is downregulated by CD63 and IL-21. J. Immunol. 186:2800–2808. 10.4049/jimmunol.1003401 21270405

[bib89] Zeng, R., R. Spolski, E. Casas, W. Zhu, D.E. Levy, and W.J. Leonard. 2007. The molecular basis of IL-21-mediated proliferation. Blood. 109:4135–4142. 10.1182/blood-2006-10-054973 17234735 PMC1885510

[bib90] Zhang, T., D.H. Kim, X. Xiao, S. Lee, Z. Gong, R. Muzumdar, V. Calabuig-Navarro, J. Yamauchi, H. Harashima, R. Wang, . 2016. FoxO1 plays an important role in regulating β-cell compensation for insulin resistance in male mice. Endocrinology. 157:1055–1070. 10.1210/en.2015-1852 26727107 PMC4769368

[bib91] Zhang, Y., L. Tech, L.A. George, A. Acs, R.E. Durrett, H. Hess, L.S.K. Walker, D.M. Tarlinton, A.L. Fletcher, A.E. Hauser, and K.-M.M. Toellner. 2018. Plasma cell output from germinal centers is regulated by signals from Tfh and stromal cells. J. Exp. Med. 215:1227–1243. 10.1084/jem.20160832 29549115 PMC5881458

[bib92] Zotos, D., J.M. Coquet, Y. Zhang, A. Light, K. D’Costa, A. Kallies, L.M. Corcoran, D.I. Godfrey, K.-M. Toellner, M.J. Smyth, . 2010. IL-21 regulates germinal center B cell differentiation and proliferation through a B cell-intrinsic mechanism. J. Exp. Med. 207:365–378. 10.1084/jem.20091777 20142430 PMC2822601

[bib93] Zotos, D., I. Quast, C.S.N. Li-Wai-Suen, C.I. McKenzie, M.J. Robinson, A. Kan, G.K. Smyth, P.D. Hodgkin, and D.M. Tarlinton. 2021. The concerted change in the distribution of cell cycle phases and zone composition in germinal centers is regulated by IL-21. Nat. Commun. 12:1–14. 10.1038/s41467-021-27477-0 34887406 PMC8660905

