# Supplementary material for: IL-21 shapes germinal center polarization via light zone B cell selection and cyclin D3 upregulation
Source: J Exp Med. 2023 Jul 19;220(10):e20221653. doi: 10.1084/jem.20221653 (PMC10355162; doi:10.1084/jem.20221653)
Supplement: SourceData F5 — is the source file for Fig. 5. [file JEM_20221653_SourceDataF5.pdf]

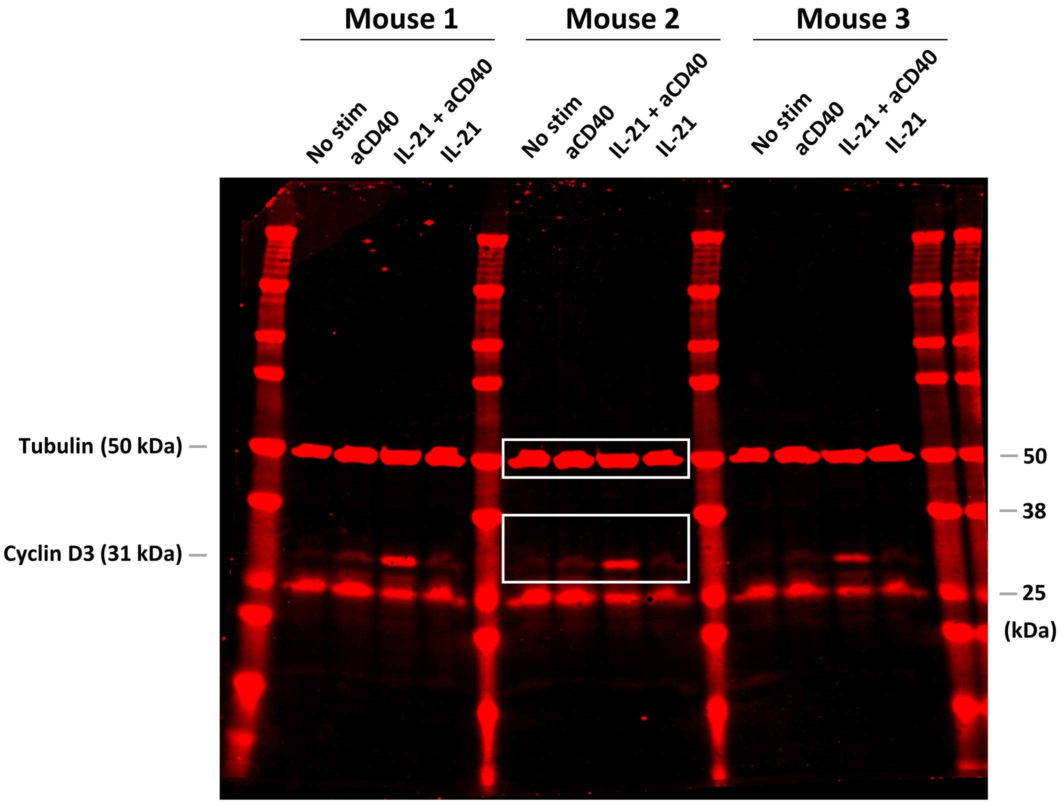

Source Data Figure 5 A

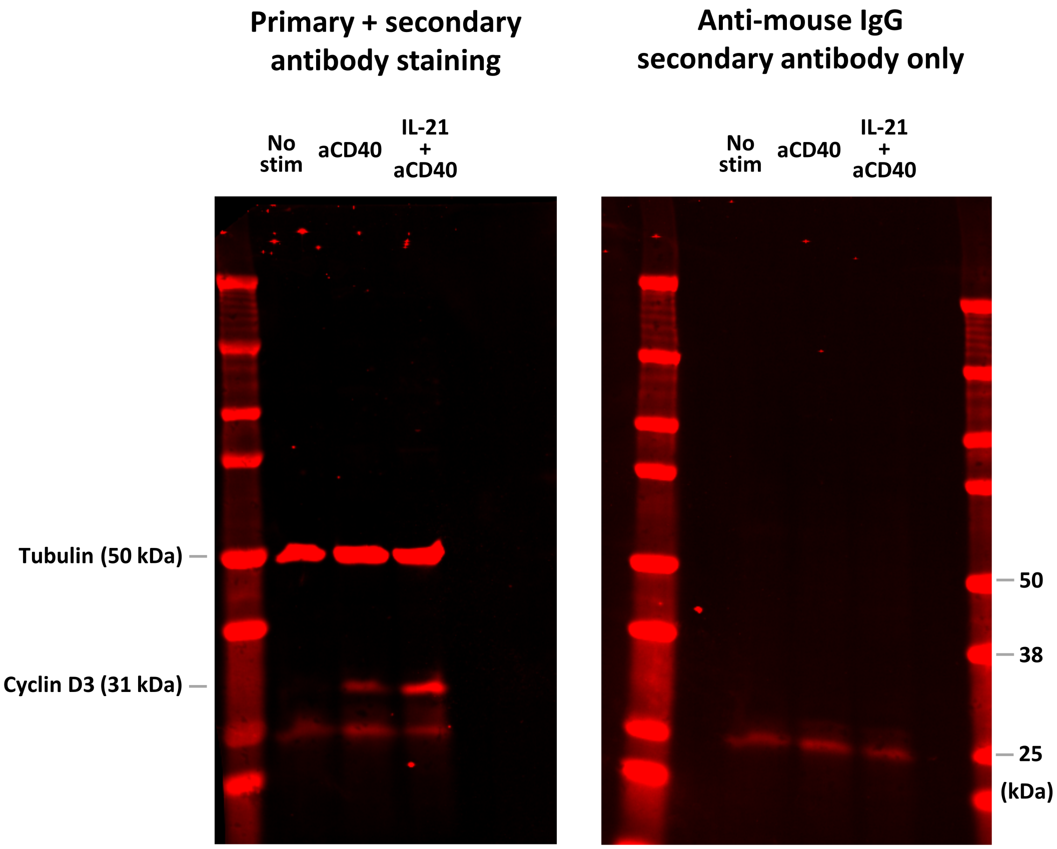

Note that the 25 kDa band appears with secondary anti-mouse IgG alone and is likely immunoglobulin light chain
